# Mechanical and Environmental Performance of Circular Concrete with Recycled Aggregates from Composite Material Waste

**DOI:** 10.1021/acsomega.6c04730

**Published:** 2026-10-01

**Authors:** Patrizia Bernardi, Federico Pagliari, Alice Sirico, Arianna Paini, Corrado Sciancalepore, Daniel Milanese, Giuseppe Vignali, Beatrice Belletti

**Affiliations:** † Department of Engineering and Architecture, 9370 University of Parma, Parco Area delle Scienze, 181/A, Parma 43124, Italy; ‡ Department of Engineering for Industrial Systems and Technologies, 703226 University of Parma, Parco Area delle Scienze, 181/A, Parma 43124, Italy

## Abstract

The construction sector has a significant environmental footprint, primarily due to its extensive use of natural resources. In the context of ongoing efforts to promote resource efficiency and reduce the consumption of nonrenewable materials, this study explores the physical, mechanical, and environmental performance of a circular concrete incorporating recycled aggregates derived from sink manufacturing waste consisting of a composite material that would otherwise be disposed of in landfills. This waste, composed of approximately one-third methacrylic resin (MMA) and two-thirds mineral filler (virgin quartz), was processed into different particle-size fractions and used as a partial replacement for both fine and coarse natural aggregates. An extensive experimental program involving more than 200 concrete specimens was carried out to assess the behavior of the developed mixtures in both fresh and hardened conditions. The investigation included the evaluation of workability, density, water absorption and permeability, mechanical performance under compressive, flexural, and tensile loading, elastic modulus, shrinkage, and postcracking response. The results indicate that the incorporation of recycled aggregates generally reduced compressive strength and stiffness, with significant reductions observed at higher replacement levels and when fine recycled fractions were used, while tensile-related properties were less affected. Overall, low replacement levels of coarse recycled aggregates provided the best balance between mechanical performance and sustainability, whereas higher replacement levels and the use of fine recycled fractions appear more suitable for nonstructural applications. The environmental performance of the different mixes was evaluated through Life Cycle Assessment. Recycled aggregates were assigned only the impacts associated with the milling process, while the impact of landfill disposal of the composite material, avoided through its valorization, was subtracted. The effect of the avoided impact is more or less significant depending on the impact category. Under the assumptions adopted in the LCA system modeling (cutoff approach with avoided landfill credit assigned to the waste material), the environmental results, referred to 1 m^3^ of concrete, demonstrate that as the percentage of replacement of natural aggregates with recycled ones increases, environmental impacts decrease. These findings support the implementation of circular economy principles in the construction sector, providing a sustainable strategy for reducing landfill waste and offering a pathway for the valorization of industrial byproducts in civil engineering applications.

## Graphical abstract

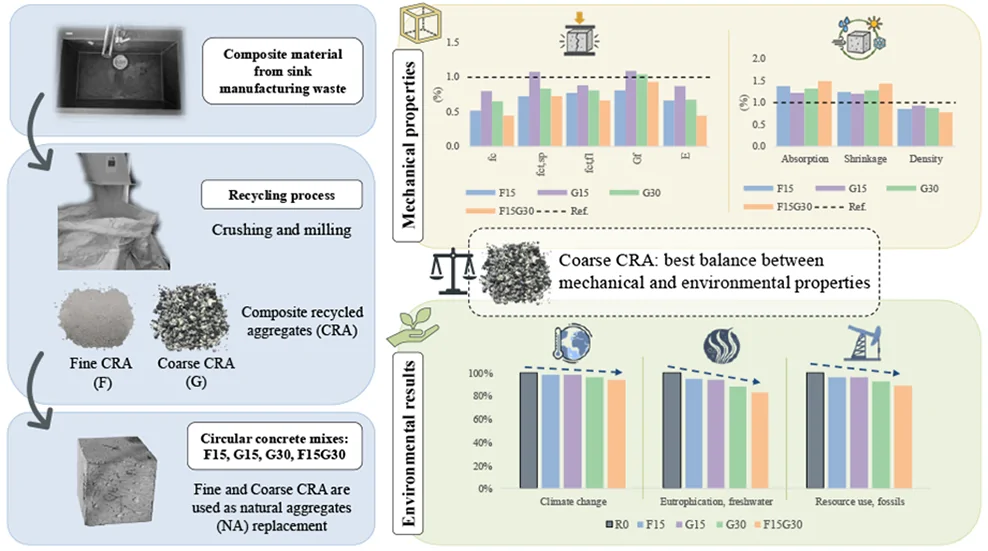

## 1 Introduction

The construction industry is widely recognized as a major contributor to environmental degradation, largely due to its extensive reliance on cement-based materials. Given that global concrete production exceeds 33 billion tons per year^1^ and is expected to increase by 30% by 2050, its environmental burden is considerable, contributing not only to greenhouse gas emissions (accounting for 8–10% of global carbon dioxide emissions)^2^ but also to the depletion of natural resources. This latter aspect is primarily linked to the large amount of natural aggregates required in concrete production, as ordinary concrete typically consists of 12% cement and 80% aggregate by mass.^3^ The extraction of sand, gravel, and crushed stone for concrete—exceeding 25 billion tons per year—results in significant environmental impacts, including landscape degradation, riverbed disruption, and widespread ecological degradation. Moreover, the mining, processing, and transportation of aggregates require considerable energy input, further exacerbating the environmental footprint of the concrete industry. In response to these challenges, considerable research efforts have focused in recent decades on the study of various types of waste as alternative components in concrete,^4,5^ with the dual objective of mitigating the aforementioned environmental impacts and addressing the increasing issue associated with solid waste management. Various types of waste materials have been investigated, including plastics,^6,7^ glass^8^ and construction and demolition waste,^9,10^ as well as residues from processing of materials of mineral origin^11–13^ or byproduct from industrial production^14^ demonstrating the potential to partially replace natural aggregates in concrete.

Among these alternatives, recycled plastic aggregates have received considerable attention due to the rapid growth in global plastic production, which increased from approximately 2 million tonnes in 1950 to 438 million tonnes in 2017,^15^ creating an urgent need for sustainable recycling solutions. Their use as concrete aggregates has therefore attracted considerable attention because of its potential to divert plastic waste from landfill, increasing the recycling rate while reducing the consumption of natural aggregates for concrete production. Numerous studies have investigated the incorporation of fully polymeric materials, such as polyethylene (PE), polypropylene (PP), polyethylene terephthalate (PET), polyvinyl chloride (PVC), and polystyrene (PS), as partial replacements for natural aggregates in concrete.^6,7^ Besides their clear environmental benefits, these materials have also been shown to improve some nonstructural properties of concrete, including thermal and acoustic insulation, while reducing its density.^16,17^ However, for structural concrete applications, several drawbacks have been reported, including significant reductions in compressive strength, elastic modulus, and, in many cases, flexural and tensile strength.^6,7,18,19^ These reductions are mainly attributed to the low stiffness and hydrophobic nature of the polymeric phase, which lead to poor adhesion with the cement paste and the formation of a weak interfacial transition zone (ITZ). Furthermore, the lower density and higher deformability of plastic aggregates also alter the stress transfer mechanisms within the cementitious matrix, leading to reduced load-bearing capacity compared with conventional concrete. Some studies^20,21^ have reported improvements in impact resistance owing to the energy-absorbing capability of polymeric inclusions, while their influence on fracture behavior has been shown to depend on several factors, including the type, geometry, size, and dosage of the plastic aggregates.^22^

Within the broader framework of reducing environmental impacts and promoting the integration of industrial processes into sustainable supply chains,^23^ this study investigates the development of more sustainable and circular concrete containing composite industrial waste. More in detail, it explores the effects on mechanical and durability performance of partially replacing natural aggregates with an industrial waste resulting from sink manufacturing processes, aligning with the current strategies aimed at promoting circular economy and enhancing waste recycling in the building sector. In addition to the experimental investigation, the environmental performance of the proposed mixtures is assessed through a Life Cycle Assessment (LCA), in order to quantify the potential benefits associated with the substitution of natural aggregates and the avoidance of landfill disposal of the composite waste. This combined approach enables a more comprehensive evaluation of the material, considering both engineering performance and environmental sustainability.

The composite material considered in this study consists of a mixture of about 65–80% mineral inorganic matrix (virgin quartz) and 20–35% organic binder (methacrylic-based resin) and is currently used for kitchen sink manufacture. Since composite molding is a complex chemical process, defective sinks can be produced. Although the scraps and offcuts generated by the sink manufacturing process are of high quality and characterized by good strength, durability, nontoxicity, resistance to heat and fire, and electrical insulation properties, they are now typically sent to landfill because of the presence of the polymeric resin.

Despite the widespread use of this type of composite material—more generally comprising quartz/granite and acrylic or polyester binder—in the manufacturing of kitchen and bathroom sinks especially in Europe, North America and Australia, but also increasingly in other international markets (such as Asia and South America), its reuse as a recycled aggregate in concrete has not yet been investigated in the scientific literature. The absence of prior studies on this specific waste material represents a research gap, which highlights the need for research at an international level. It is important to highlight that the composite waste investigated in this study differs substantially from the conventional plastic aggregates commonly examined in previous research. Unlike fully polymeric materials such as PE, PP, PET, and PVC, which are characterized by low stiffness, high deformability, and negligible mineral content, the investigated material is a hybrid composite with a dominant inorganic quartz phase bound by a methacrylic-based resin. As a result, its density, stiffness, hardness, thermal stability and microstructural morphology differ markedly from those of traditional plastic aggregates. These distinctions suggest that the interaction mechanisms with the cementitious matrix—particularly at the interfacial transition zone (ITZ)—may not follow those typically reported for fully polymer-based aggregates. In this sense, understanding whether the behavior of this composite waste aligns with that of plastic aggregates, mineral aggregates, or exhibits an intermediate or entirely distinct response represents a scientific question that has not yet been addressed in the literature. In this context, this research aims to assess the influence of two different particle size distributions—one finer and one coarser—on key concrete properties, including density, workability, elastic modulus, shrinkage, compressive, flexural and tensile strength, as well as postcracking behavior, while also assessing the environmental performance of the proposed mixtures through LCA.

## 2 Recycled Aggregates from Composite Material Waste

### 2.1 Physical Properties

The waste material used in this experimentation was provided by an Italian company that is a leader in the production of composite sinks. More in detail, it derives from discarded items with aesthetic imperfections as well as from production residues, such as the trimmings removed from the edges of sinks, drains, overflows, and residues from cutting operations. As a result, the waste can exhibit a wide range of shapes and sizes. Although siliceous and silicate materials (quartz and alkali and alkaline-earth metal silicates) are the main components of the material, the presence of an acrylic-based resin—accounting for approximately 30% of the total and being inseparable from the composite—makes landfill disposal currently the only viable option for this type of waste. Moreover, due to the industrial production process, the presence of other minor compounds, such as glass fibers and silicon-based minerals, cannot be excluded.

The composite material waste was first ground using a dedicated plant located within the company itself and was supplied to be used as recycled aggregate in two different grain sizes, i.e., fine (approximately 0.1–2 mm), and coarse (1–8 mm), Figure 1a and Figure 1b, respectively. The grain size distribution of the two composite recycled aggregate (CRA) typologies, in terms of squared mesh size versus cumulated sieve passing, was obtained according to specifications of EN 933–1:2012 ^24^ for virgin aggregates and is reported in Table 1. Water absorption according to EN 1097–6:2022 ^25^ was found to be equal to 0.025% (std. dev. 0.01) for both fine and coarse composite recycled aggregates, while density, measured according to EN 1097–6:2022,^25^ was equal to 2.003 g/cm^3^ for the fine material (std. dev. 0.009) and 1.887 g/cm^3^ for the coarse material (std. dev. 0.005).

**Figure 1:**
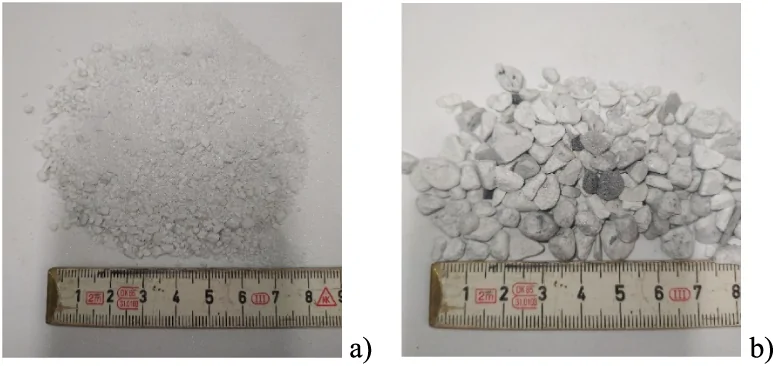
Composite recycled aggregates as supplied by the manufacturer: (a) fine and (b) coarse.

**Table 1:** Particle Distribution of Composite Recycled Aggregate (CRA) Used in the Concrete Mix

	**Cumulative** **passing [%]**	
**Sieve (mm)**	**Coarse CRA**	**Fine CRA**
10.000	100.00	100.00
8.000	97.62	100.00
6.300	70.79	100.00
4.750	34.59	100.00
4.000	24.91	99.88
2.000	7.65	94.06
1.000	1.70	78.53
0.500	0.59	60.30
0.250	0.39	23.95
0.125	0.28	5.73
0.063	0.18	1.37

### 2.2 Chemical Properties

#### 2.2.1 Infrared Spectroscopy

CRA is subjected to Fourier transform-infrared spectroscopic (FT-IR) characterization to determine the molecular structure of components, in Figure 2.

**Figure 2:**
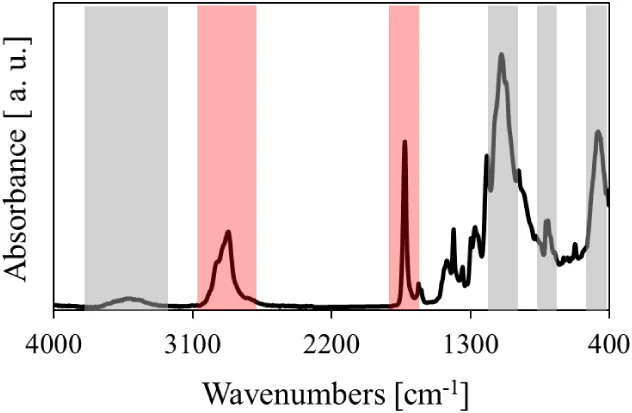
FT-IR spectrum of CRA.

The bands at 470, 800, and 1097 cm^–1^ can be attributed to the bending vibration, symmetric and asymmetric stretching vibration of the Si–O–Si bond, respectively. The broad band around 3300–3700 cm^–1^ is caused by the stretching vibration of −OH group.^26^ The absorption bands of the inorganic silicate fraction are shown in gray in Figure 2. The absorption peaks at 2950, 2870, and 1724 cm^–1^ are attributable respectively to the asymmetrical and symmetrical stretching of the −CH_3_ groups and to the stretching of the C=O groups,^27^ typical characteristics of acrylic-based thermosetting resins and highlighted in red in Figure 2. Therefore, the absorptions of the inorganic and organic parts of the composite material are both clearly visible.

#### 2.2.2 Electron Microscopy and Elemental Analysis

The geometric features of CRA were analyzed using scanning electron microscopy (SEM). In Figure 3a, the aggregates are seen to consist of highly irregularly shaped granules with heterogeneous composition, having dimensions even smaller than a millimeter. The material heterogeneity is also highlighted by the presence of a fibrous component, shown in Figure 3b. The different phases are emphasized by the different image contrast, due to the acquisition using backscattered electrons: the lighter parts are those composed mainly of silicon, while the darker areas have a carbon-based composition. The compositional elemental analysis of the waste material was determined by the energy dispersion spectroscopy (EDS). The simultaneous presence of carbon signal (C), from the acrylic component, and of silicon signal (Si), from the silicate inorganic fraction, can be noted in the EDS spectrum in Figure 3c, confirming what was observed in the SEM image.

**Figure 3:**
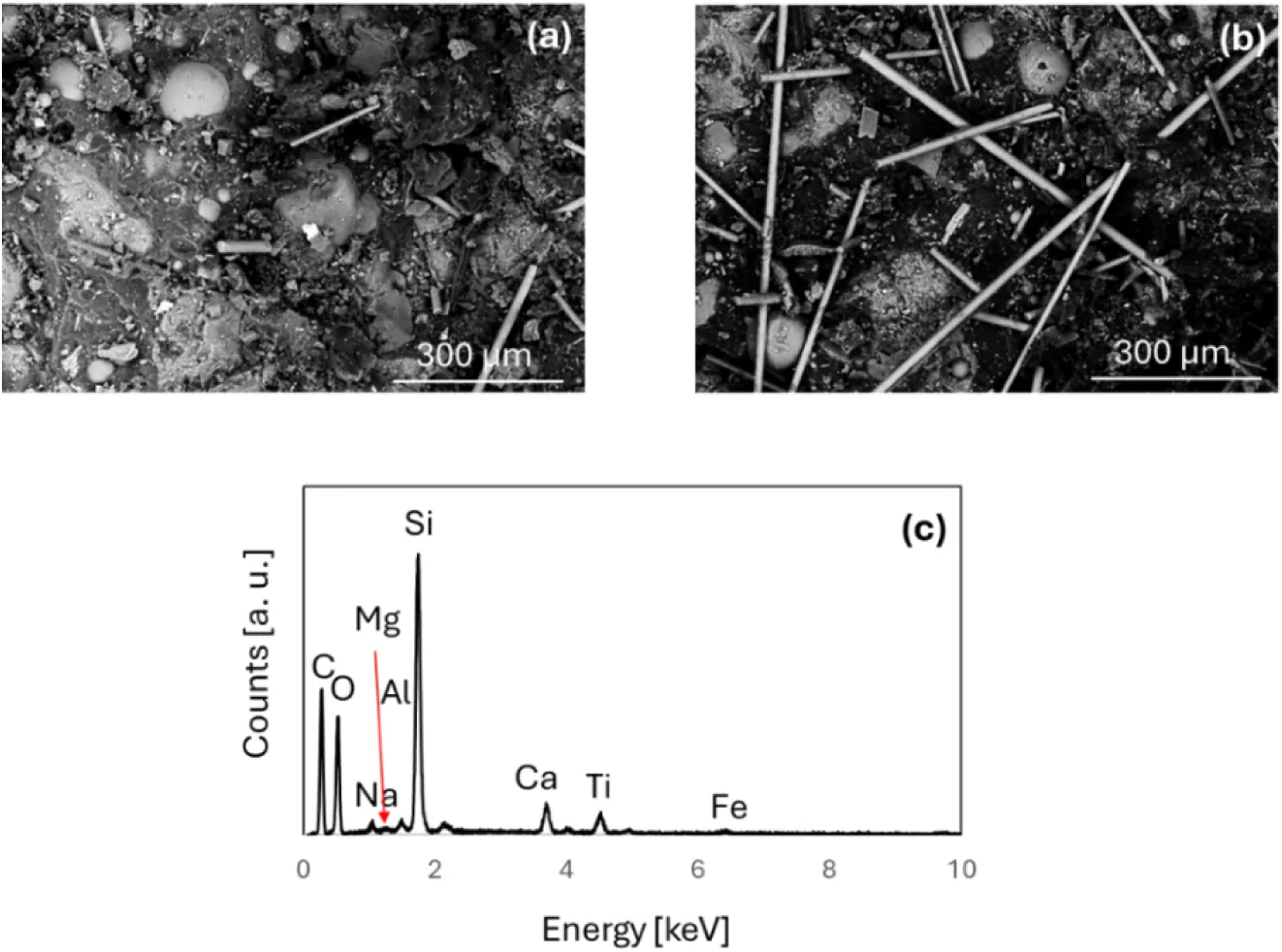
(a, b) SEM images, and (c) EDS spectrum of CRA.

#### 2.2.3 Leaching Test

To assess waste safety for its use in concrete mix, pH measurement, as well as heavy metals and anions concentration were determined. The pH value of 7.5 confirms the total not harmfulness of the waste.

The test for the evaluation of heavy metals concentration showed that all the compounds analyzed were below the limit of detection, except for Nickel, which presented a value of 1 μg/l. Anyway, this value is under the established threshold of environmental biosafety, which is equal to 10 μg/l.

The release of chlorides, cyanides, fluorides, nitrates, and sulfates was under the limit of detection for all the anions.

Hence, by considering the results of the leaching test, the composite waste material investigated in this study was considered safe for the following experiments as well as for its use in the building industry, since it does not pose risks for environmental and human health at all.

## 3 Experimental Program

### 3.1 Mix Design and Specimen Casting

The experimental campaign involved five concrete mixtures, including a reference mix with only natural aggregates (NA) for control (R0), representative of a conventional concrete typically employed in standard structural applications, a concrete mix containing 15% of fine (F15), two concrete mixes containing an increasing percentage of coarse (G15 and G30) and a mix with both fine and coarse composite recycled aggregates (CRA) in a proportion of 15% and 30% respectively (F15G30).

Portland Limestone Cement Type CEM II/A-LL 32.5 R, siliceous sand and gravel were used for all the concrete mixes. Regarding the physical properties of natural aggregates, the particle density and the water absorption (defined as the water content of an aggregate in saturated but surface dry condition—SSD), measured according to EN 1097–6:2022,^25^ were equal to 2.683 g/cm^3^ and 0.90% for sand and to 2.632 g/cm^3^ and 0.89% for gravel.

Table 2 reports the nominal mix proportions, defined according to a volumetric design approach, where both natural aggregates (NA) and composite recycled aggregates (CRA) are expressed in saturated surface-dry (SSD) condition. A constant effective water-to-cement ratio of 0.5, as well as a constant superplasticizer (SP) amount, were adopted for all the mixtures, while the aggregate substitution ratios, expressed as percentages of the total aggregate volume in each mixture, were varied among the mixes. Accordingly, the total mixing water was adjusted to reflect the measured water absorption of each NA/CRA fraction and to compensate for SSD corrections, thereby ensuring that the effective water-to-cement ratio (equal to 0.50) and the paste volume remained constant across all mixtures.

**Table 2:** Mix Proportions of the Concrete Specimens (NA: Natural Aggregates; CRA: Composite Recycled Aggregates; SP: Superplasticizer)

	**Components [kg/m** ^**3**^]						
**Mix**	**Fine NA**	**Coarse NA**	**Cement**	**Fine CRA**	**Coarse CRA**	**Water**	**SP**
R0	583.42	1188.20	400	-	-	200	1.10
F15	346.21	1103.42	400	193.41	-	200	1.10
G15	518.99	933.21	400	-	182.77	200	1.10
G30	518.99	678.56	400	-	365.55	200	1.10
F15G30	346.21	593.78	400	193.41	365.55	200	1.10

As can be seen in Figure 4, the criterion by which the replacement percentages were chosen for the development of the experimental mixtures was to obtain granulometric fuses comparable with the reference one, which in turn was chosen to be as close as possible to that suggested in the standard EN 1766:2000.^28^

**Figure 4:**
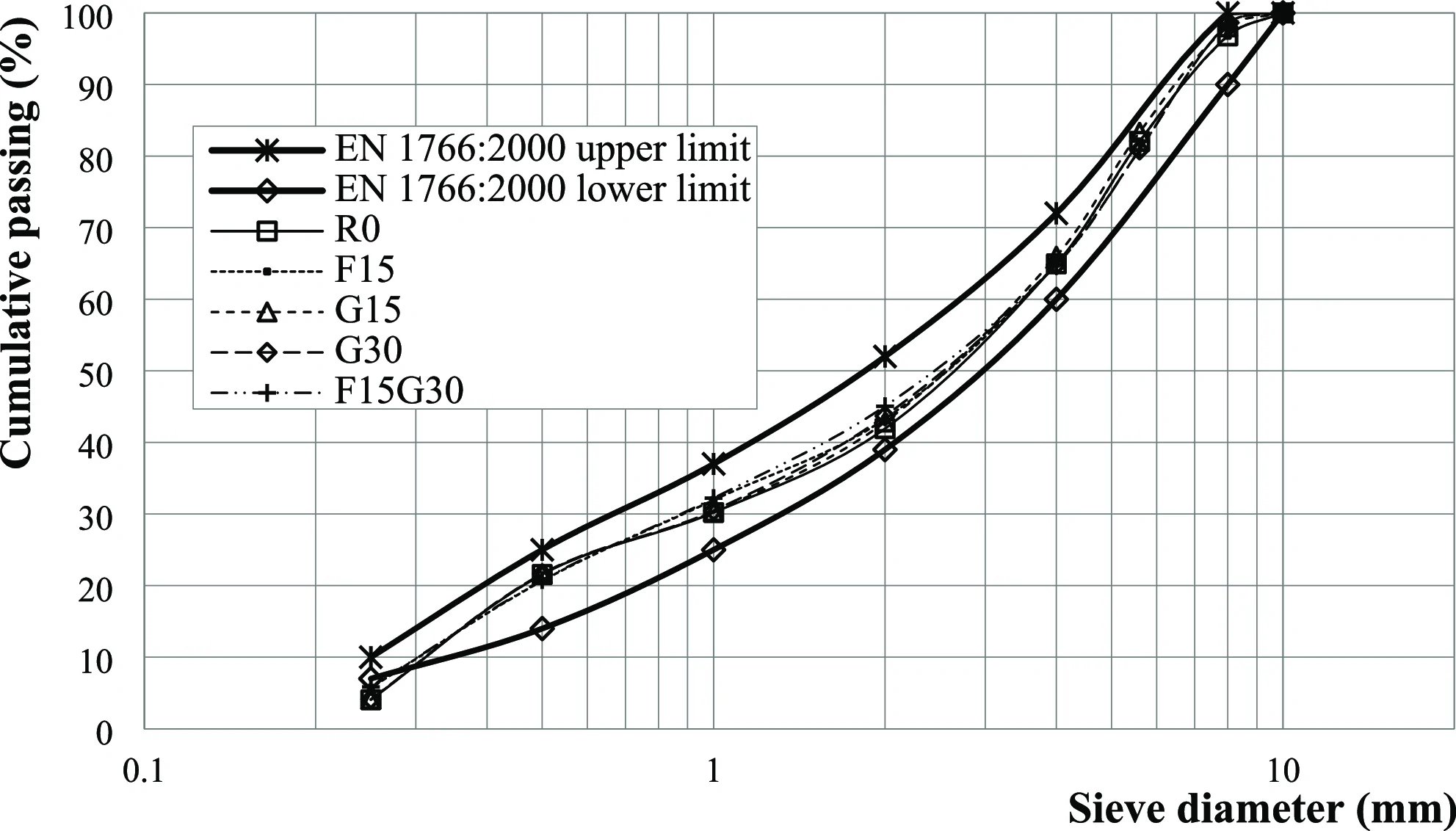
Particle distribution of concrete mixes with composite recycled aggregates compared to reference concrete mix.

All mixtures were prepared to ensure homogeneity and prevent any risk of segregation during casting. Initially, half of the aggregates (both natural and recycled, when used) were mixed in a drum-type mixer for about 2 min with about a quarter of the total mixing water. Then, the remaining aggregates and another quarter of the water were introduced and mixed for additional 2 min. Cement and the remaining water were then added and mixed for 6 min, stopping briefly after 3 min to scrape off any concrete adhering to the mixer. At the same time, the superplasticizer was incorporated into the admixture as a solution in the remaining portion of the mixing water.

Cylindrical (100 × 200 mm, *d_c_* × *h*_*c*_), cubic (100 × 100 × 100 mm), and prismatic (100 × 100 × 400 mm and 40 × 40 × 160 mm) specimens were cast to perform splitting, compressive, flexural and fracture energy tests and to measure elastic modulus and shrinkage. Additional specimens (150 × 150 × 150 mm cubes and 100 × 50 mm, *d_c_* × *h*_*c*_, cylinders) were prepared for density, water absorption, depth of penetration of water under pressure and water sorptivity measurements. To prevent moisture loss, after casting, all the specimens were protected with polyethylene sheets until demolding, which was carried out after 24 h. Water curing was adopted for all the specimens, except for the cubes intended for density and water absorption measurements, whose curing followed the specific requirements set by the relevant testing standards (EN 12390–7:2019 ^29^ and ASTM C642–06 ^30^ respectively).

The overview of the experimental campaign is reported in Table 3.

**Table 3:** Experimental Campaign Overview

**Test type**	**Number of specimens per test type per mix**	**Specimen** **dimensions [mm]**	**Reference standard**
Slump	3	-	EN 12350–2:2019
Fresh density	3	-	EN 12350–6:2019
Hardened density	3	150 × 150 × 150	EN 12390–7:2019
Water absorption	3	150 × 150 × 150	ASTM C642–06
Sorptivity	3	Φ100 × 50	ASTM C1585–13
Depth of penetration of water under pressure	3	150 × 150 × 150	EN 12390–8:2019
Drying shrinkage	3	40 × 40 × 160	EN 12390–16:2019
Compressive strength 7 days	5	100 × 100 × 100	EN 12390–3:2019
Compressive strength 28 days	5	100 × 100 × 100	EN 12390–3:2019
Compressive strength 120 days	5	100 × 100 × 100	EN 12390–3:2019
Splitting tensile strength	4	Φ100 × 200	EN 12390–6:2009
Flexural strength	3	100 × 100 × 400	JCI-S-001–2003
Fracture energy	3	100 × 100 × 400	JCI-S-001–2003
Elastic modulus	6	40 × 40 × 160	EN 12390–13:2021

### 3.2 Workability and Density Tests

According to EN 12350–2:2019,^31^ slump test was used to assess workability.

The fresh density was evaluated in accordance with EN 12350–6:2019,^32^ following compaction of the concrete using a vibrating needle. Hardened concrete density was determined 28 days after demolding, according to EN 12390–7:2019,^29^ on three cubic specimens for each mix. The cubes were dried at 105 ± 5 °C in a ventilated oven until their mass variation in two successive weightings (at intervals of 24 h) was less than 0.2%.

### 3.3 Durability Tests: Water Absorption, Sorptivity, Depth of Penetration of Water under Pressure, and Shrinkage

To provide a preliminary assessment of the durability-related performance of concrete containing composite recycled aggregates, tests focusing on moisture transport properties and drying shrinkage were conducted. Although these tests do not constitute a comprehensive evaluation of the long-term durability of the investigated concretes, they provide useful information on moisture transport behavior and dimensional stability, which are key parameters influencing durability performance.

The ability of concrete containing this type of recycled aggregates to absorb water was first evaluated in terms of total amount of water absorption, by using the saturation method described in ASTM C642–06 ^30^ on three cubes for each batch. For each specimen, the oven-dry mass (A) was obtained by drying it in a ventilated oven at 105 ± 5 °C until an approximately constant mass was achieved, defined as a mass variation of less than 0.5% in 24 h. The saturated mass (B) of the samples was then obtained by water immersion at approximately 20 °C for no less than 48 h and until the mass variation, measured on surface-dried specimens, was below 0.5%. Water absorption was then calculated with the expression: [(B–A)/A] × 100.

The rate of absorption (sorptivity) of water was measured according to ASTM C1585–13 ^33^ by adopting three cylindrical specimens with a 100 mm diameter and a height of 50 mm for each concrete admixture. The tests were performed by measuring the increase in mass of the specimens resulting from absorption of water as a function of time, when only one surface of the specimen is exposed to water. At first, the specimens were conditioned by oven-drying at a temperature of 50 °C for 3 days; then, an internal relative humidity of about 50% was reached by storing them in a sealed container at 20 °C for 20 days, to avoid a moisture gradient in concrete depth. Before test, the side surface of each specimen was sealed with an epoxy resin coating, while a removable plastic layer was used to cover the upper surface and prevent water evaporation. The samples were put in a plastic container on stainless-steel rods, where the water level was maintained 2 mm above the top of the supports for the whole test duration, allowing continuous water exposure for the lower surface. The mass of the specimens was measured at predefined intervals according to ASTM C1585–13 ^33^ up to 10 days. Then, the absorption was calculated as the ratio between the mass variation (in grams) during testing and the product between the exposed area of the sample (i.e., the base, in mm^2^), and the density of water (in g/mm^3^). Therefore, absorption is expressed in mm.

Depth of penetration of water under pressure was measured on three cubes for each batch according to the procedure indicated in EN 12390–8:2019:^34^ after conditioning, a water pressure of 500 kPa was applied on a time span of 72 h on the lower surface of the specimen by means of a testing apparatus able to maintain constant pressure. After the specified time, the specimens were split in half perpendicularly to the face on which the pressure was applied. After a little drying, the depth of penetration of water (expressed in mm) was measured on the split surface of the specimens.

Shrinkage tests were performed on three 40 × 40 × 160 mm specimens for each batch, according to EN 12390–16:2019.^35^ After demolding, the specimens were stored under laboratory conditions (average ambient temperature: 22 ± 2 °C; average relative humidity: 68 ± 6%), with ambient temperature and relative humidity monitored and recorded once daily throughout the test period. Free linear shrinkage was determined by measuring the length variation of the specimen along the longitudinal axis by using a digital dial comparator, which was characterized by a 0.001 mm subdivision, at predefined intervals for 56 days after demolding.

### 3.4 Mechanical Tests

Compressive strength tests were conducted in accordance with EN 12390–3:2019 ^36^ after 7, 28, and 120 days of curing, by using a Universal Testing Machine METROCOM PV P30. The measurements were carried out on five cubic specimens for each concrete mix, at each time of curing; a constant rate of 0.5 MPa/s was used for load application.

Splitting tensile tests were performed on four cylindrical specimens using the same Universal Testing Machine, following EN 12390–6:2009 ^37^ recommendations. With the specimen positioned horizontally between the plates of the testing machine, a compressive load was increased continuously at a constant rate of 0.05 MPa/s along the length of the cylinder until the brittle failure of the specimen occurred.

Flexural tests were carried out employing a three-point bending (3PB) load configuration, following the procedure exposed in JCI-S-001–2003.^38^ The beam specimens were prenotched at midspan with a cut measuring 2 mm in width and 30 mm in depth. Three samples for each mix were tested using a high-precision Instron 8862 servo-electric Universal Testing Machine, by controlling the crack mouth opening displacement (CMOD) at a constant rate of 3 mm/h with a clip-on strain gauge, with simultaneous load measurement. Flexural strength σ_*f*_ and fracture energy *G*_*f*_ were calculated from the load P-CMOD response, according to eqs 1 and 2, respectively:

1 $$\sigma_{f}=P_{max}\frac{3S}{2bh^{2}}$$

2 $$G_{f}=\frac{0.75W_{0}+W_{1}}{A_{lig}}$$

where *b* and *h* are the width and net depth of the notched midcross section (which are equal to 100 mm and 70 mm, respectively), while *S* represents the net span of the specimen (equal to 300 mm). *P*_*max*_ and *W*_0_ indicate respectively the peak load and the area under P-CMOD curve, while *W*_1_ is related to the work done by the dead weight of the beam and the loading equipment, and *A*_*lig*_ is the area of the ligament (equal to *b* × *h*).

Elastic modulus tests were performed according to EN 12390–13:2021 (Method A)^39^ on a batch of six specimens for each concrete admixture: three preload cycles were performed to verify the stability and the positioning of the specimen, ranging between a baseline stress (σ_*p*_) of 0.5 MPa and an upper stress of 0.1–0.2 times the value of the compressive strength of the specimen (σ_*b*_), at a loading rate of (0.6 ± 0.2) MPa/s. Both loads in each cycle were applied within a time span not exceeding 20 s. Then, three loading cycles were performed: for each cycle, the stress was increased from the lower value σ_*b*_ at a rate of (0.6 ± 0.2) MPa/s up to the upper stress *σ*_*a*_ (equal to one-third of the compressive strength of the specimen). Again, both loads in each cycle were applied within a time span not exceeding 20 s. At the end of the upper stress phase of the first cycle, when the load is stable, the corresponding strains along each measurement line were recorded and the average strains *ε*_*a,1*_ at these stress levels were calculated. At the end of the third cycle, the compressive strength of the specimen was determined by loading the specimen until failure.

The initial secant modulus of elasticity is then calculated as:

3 $$E_{C,0}=\frac{\Delta \sigma }{\Delta \varepsilon_{0}}=\frac{\sigma_{a}^{m}-\sigma_{b}^{m}}{\varepsilon_{a,1}{‐\varepsilon }_{b,0}}$$

where ε_b,0_ represents the strain corresponding to the stress level σ_b_ previously described, and σ_b_^m^ and σ_a_^m^ represent, respectively, the measured lower and upper stress values measured during the loading cycles prior to the specimen failure.

## 4 Results

### 4.1 Workability and Density

The results of slump tests are reported in Figure 5: as can be seen, the workability is not significantly affected by the presence of composite recycled aggregates, and the values obtained are comparable with the value of the reference mix. The maximum difference was observed for G30 mixture, which shows an increase in the slump value of approximately 7%.

**Figure 5:**
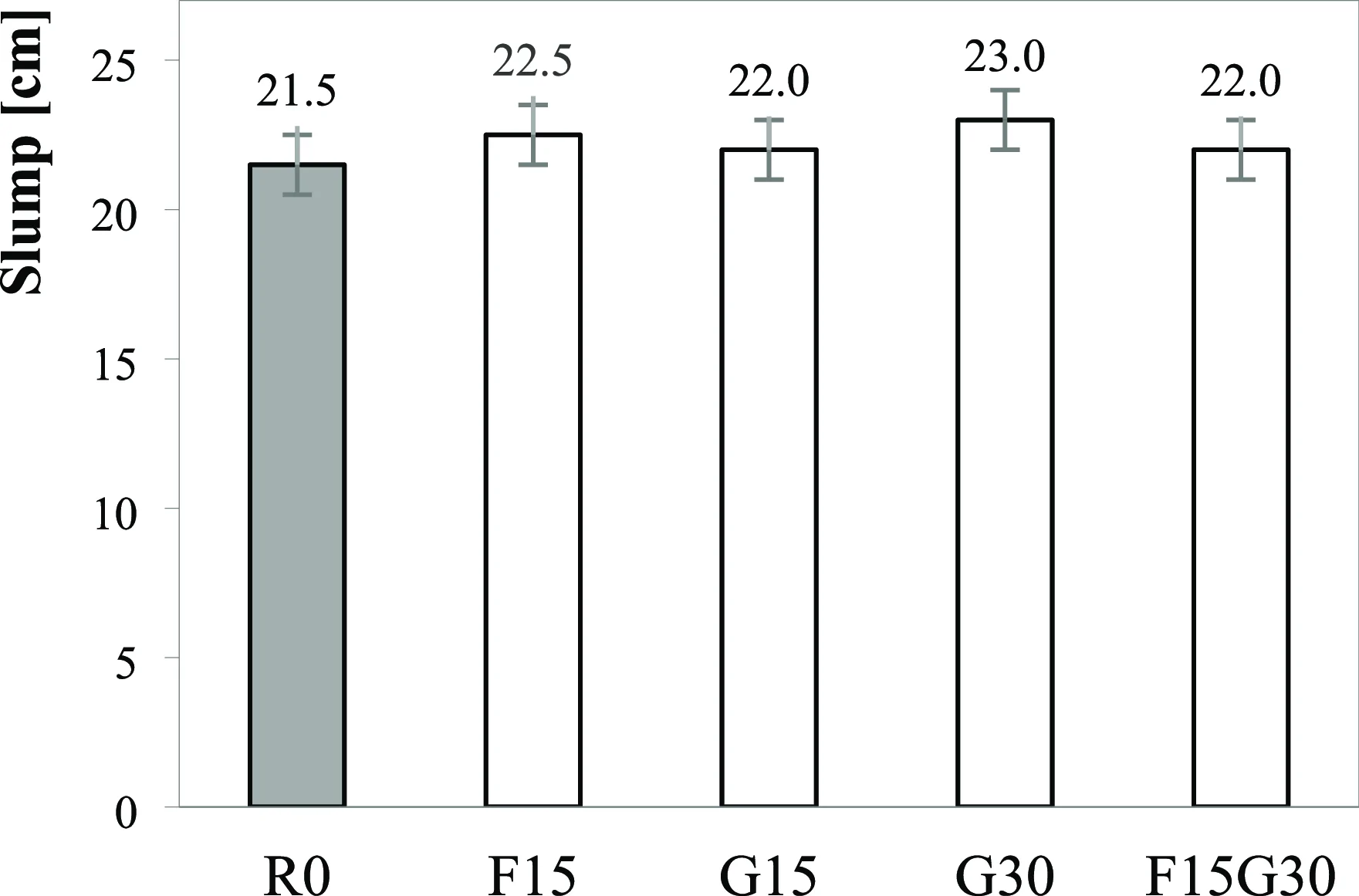
Slump test results.

The effect of composite recycled aggregates on density can be seen in Figure 6. By comparing the obtained values with those of the reference natural aggregate (NA) concrete (R0), it is evident that the inclusion of CRA results in a decrease in density, following the same trend in the measurements both in the fresh and hardened state, due to the approximately 30% lower density of composite recycled aggregates (both fine and coarse) with respect to natural ones as well as increased porosity in concrete. More in detail, the increased porosity is mainly associated with the interfacial transition zone (ITZ) between the cement paste and the composite recycled aggregates. As it will be discussed in detail in Section 6, the hydrophobic polymeric fraction of the CRA limits its adhesion to the cementitious matrix, promoting the formation of localized interfacial voids that contribute to the increase of the overall pore volume of the concrete. Consequently, the reduction in density is more pronounced when fine CRA are introduced, either alone or in combination with coarse CRA, because the larger total ITZ area associated with fine particles increases the overall volume of weak interfaces within the concrete. Specifically, in the cases of mixtures F15 and F15G30, density reductions in the hardened condition of approximately 14% and 22% are observed, respectively. It is not surprising that the latter also represents the highest measured value, being F15G30 the mixture with the highest absolute replacement percentage among those produced. However, the density-reducing effect, although less pronounced, is also observable when the replacement involves only coarse aggregates. Mixtures G15 and G30 show density reductions in the hardened state of 7% and 13%, respectively.

**Figure 6:**
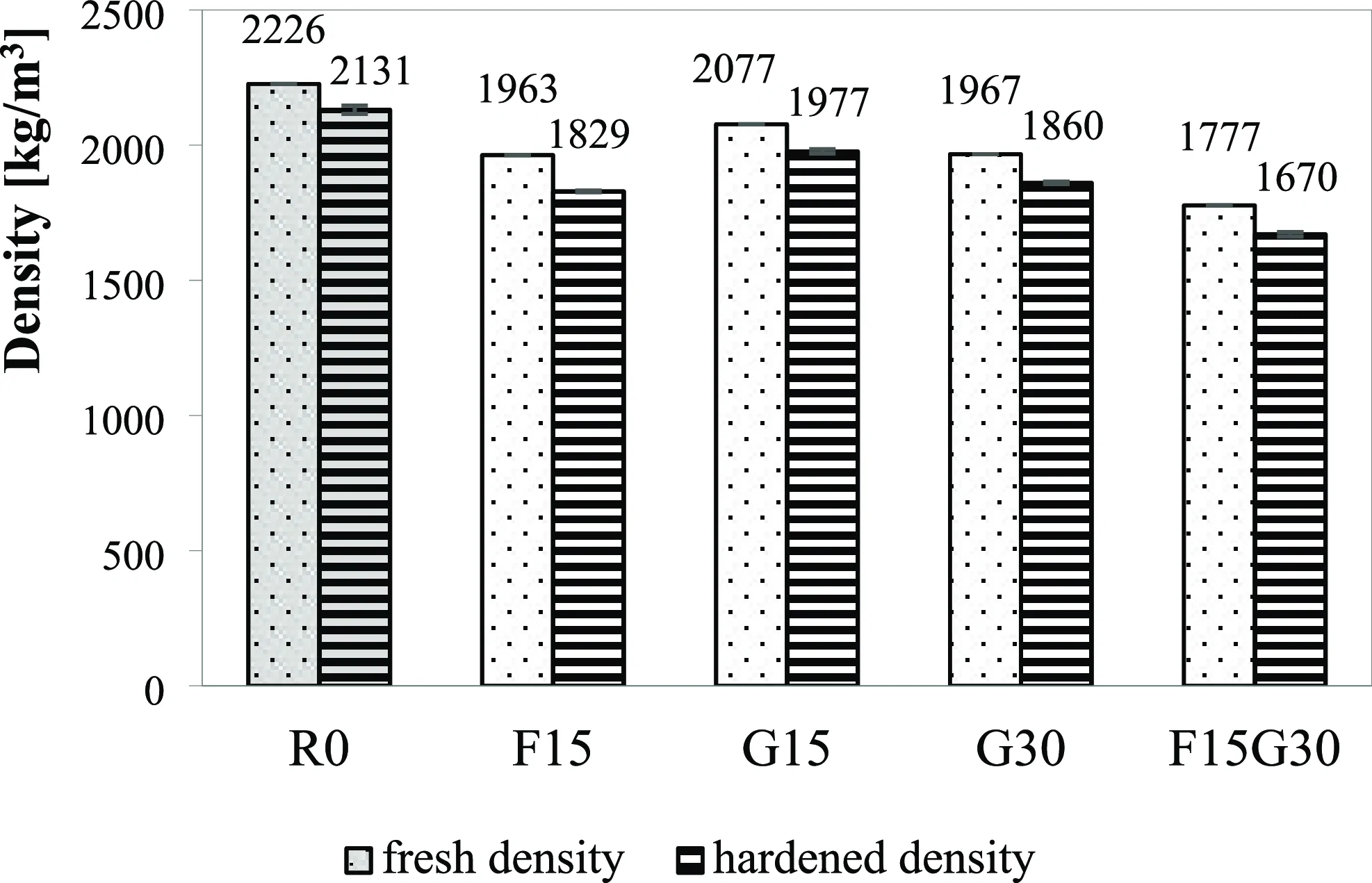
Average fresh and hardened density values with the corresponding standard deviation, for concrete mixes containing coarse and fine composite recycled aggregates.

### 4.2 Water Absorption, Water Sorptivity, and Depth of Penetration of Water under Pressure

Figure 7 shows the total water absorption values for concrete with different amounts of aggregate substitution. The test aims to saturate nearly the entire open pore network, including large, medium, and small capillary pores, to measure the total open porosity accessible to water. Although the water absorption of composite recycled aggregates themselves is less with respect to that of natural aggregates, increasing the substitution rate results in higher overall water absorption for CRA concrete compared with the reference one (R0). This phenomenon is linked to increased porosity of CRA concrete, which is primarily associated with the larger volume of weak ITZ generated around the composite recycled aggregates, where localized interfacial voids may develop because of the limited compatibility between the polymeric fraction and the cement paste. Accordingly, the effect is more pronounced when fine CRA are used, owing to the substantially larger total ITZ area associated with fine particles, consistently with the density results discussed in Section 4.1.

**Figure 7:**
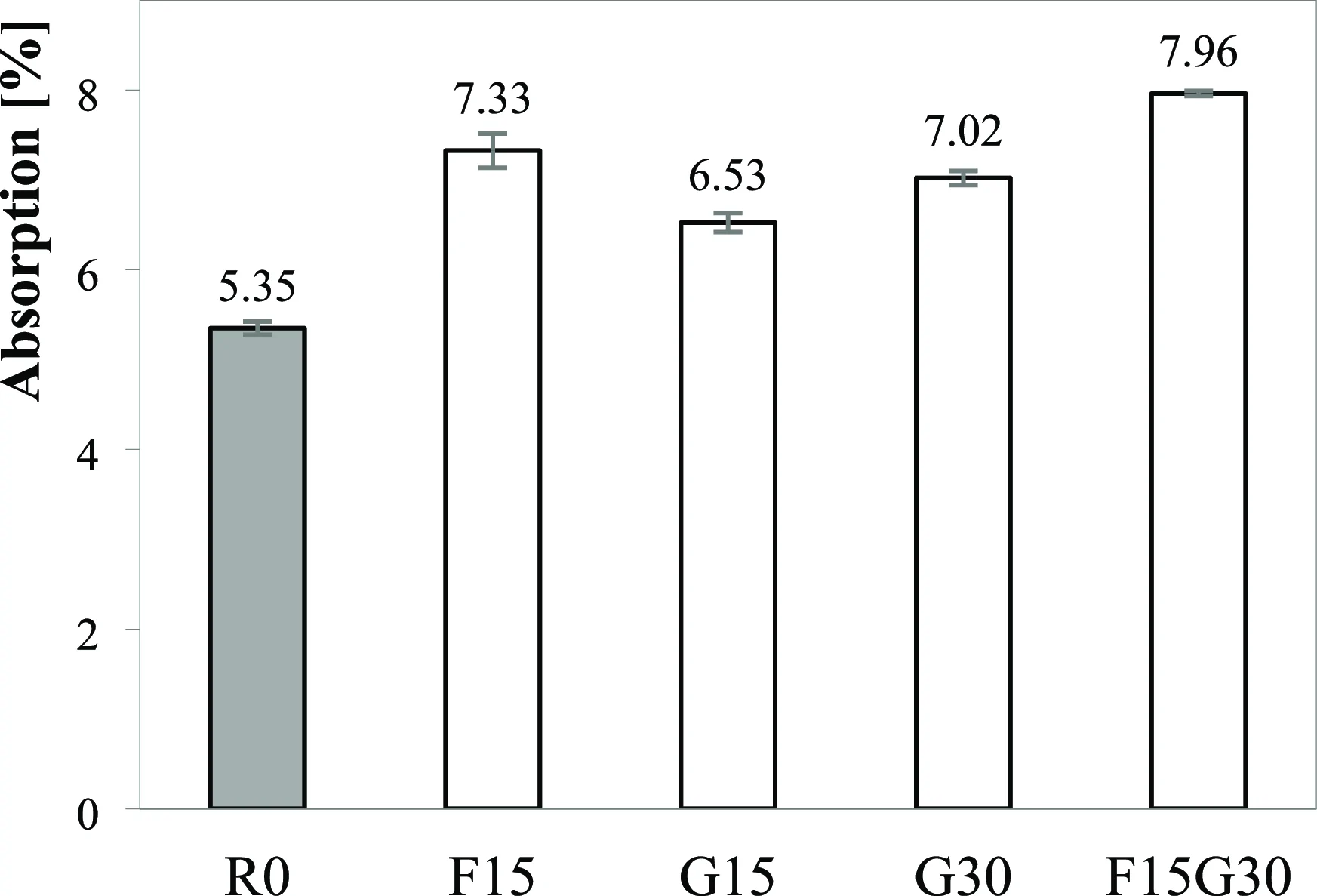
Total water absorption results.

Anyway, for all mixes, the absorption value (%) does not exceed the recommended limit for good quality cementitious materials in practical applications, which is set at 10%.^40^

Figure 8 shows the data related to the depth of penetration of water under pressure tests: it measures the interconnected porosity aligned along the pressure gradient, including microcracks and continuous networks of interconnected pores. It can be observed that the measured values increase as the substitution percentage increases, with the maximum value observed for F15G30 mix, confirming the findings already observed for total water absorption. The higher penetration depths are therefore consistent with the more porous interfacial microstructure observed in concretes containing CRA with the SEM analyses and presented in Section 6. Anyway, all the investigated concretes show values below 50 mm, which represents the maximum penetration by EHE-08 for structural concretes under XS1 environment.^41,42^

**Figure 8:**
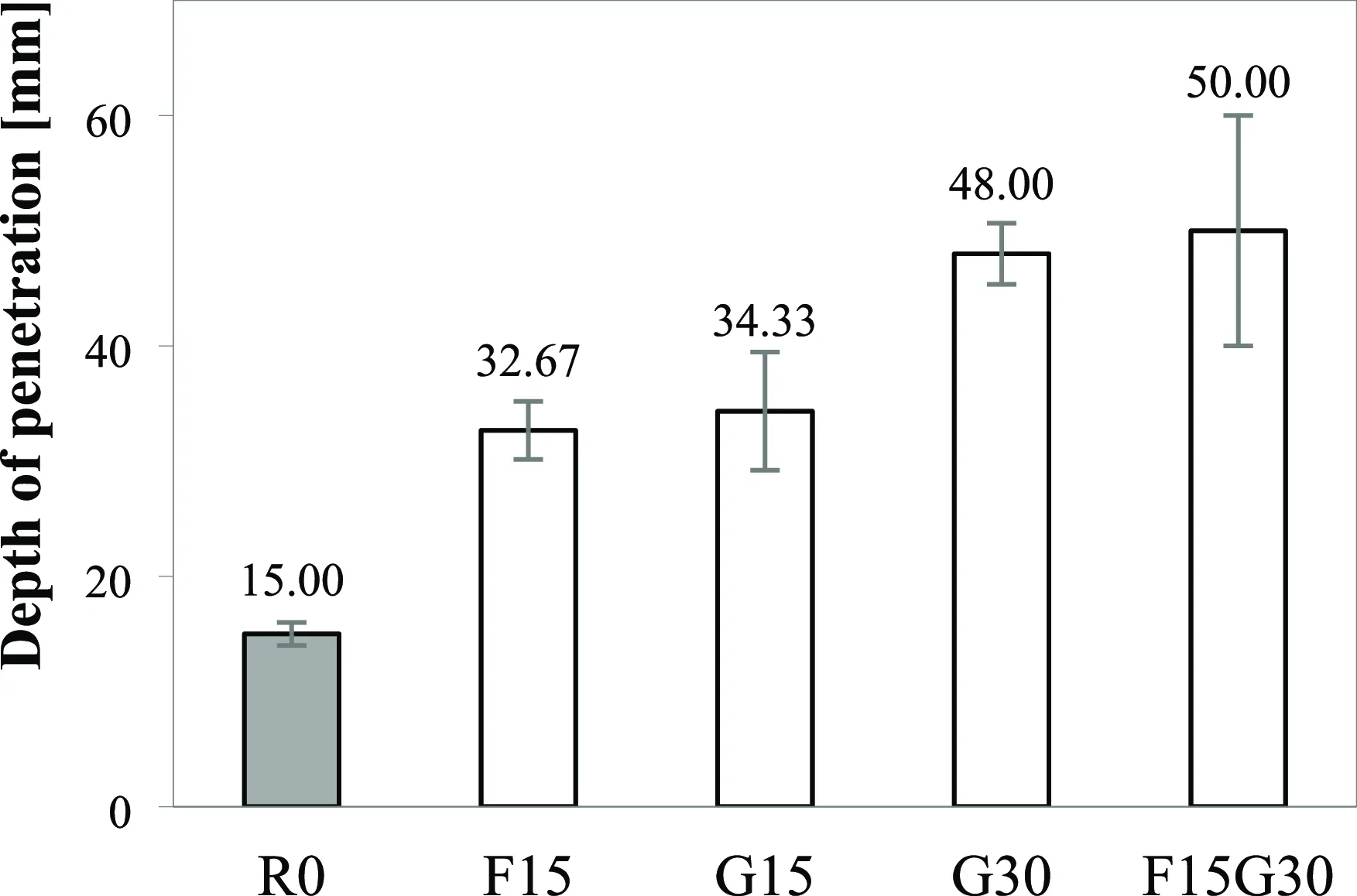
Depth of penetration of water under pressure.

Regarding sorptivity tests, the aim is to evaluate how the capillary porous microstructure of the material influences the penetration rate of aggressive agents in a real environment. The obtained absorption curves show that the behavior of the tested concrete mixtures in terms of absorption rate is almost comparable for all the mixes, both for short-term—evaluated during the first six hours of testing (Figure 9b)—and for long-term behavior (Figure 9a).

**Figure 9:**
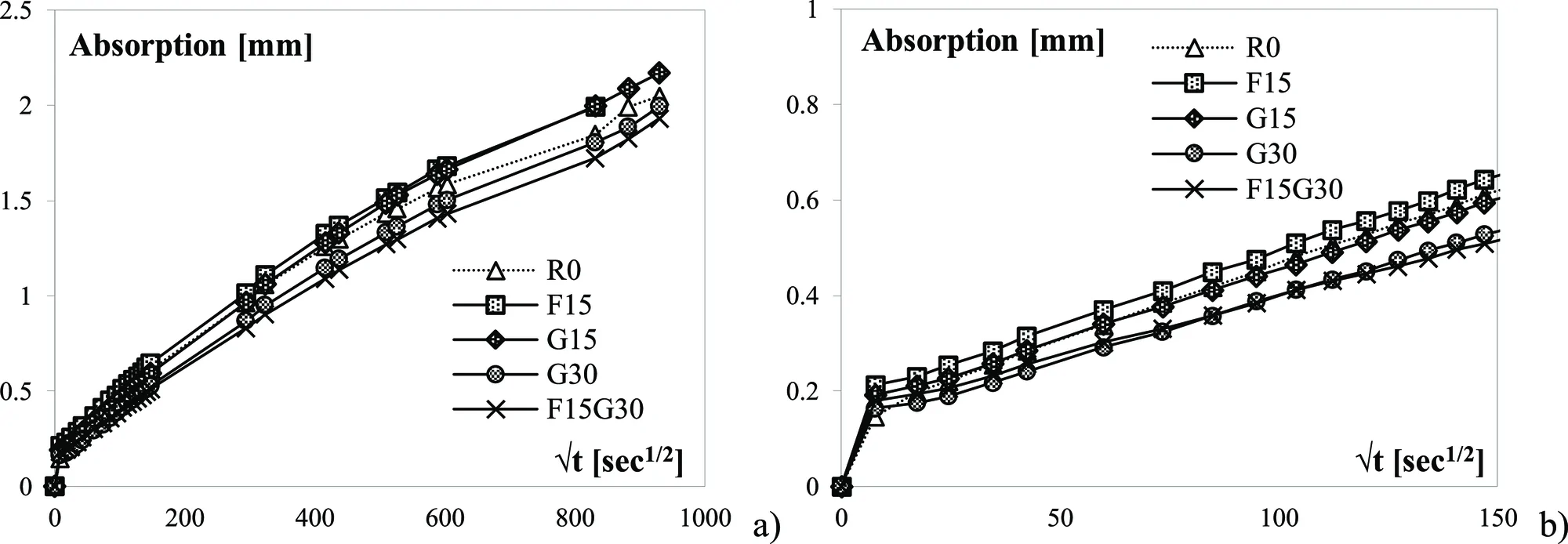
Water sorptivity test results: (a) long-term and (b) short-term evaluated during the first six hours of testing.

### 4.3 Shrinkage Tests

As shown in Figures 10 and 11, the maximum shrinkage values measured for the mixtures containing composite recycled aggregates are higher than those of the reference concrete. As can be observed from the curves in Figure 10, for all mixes, most of the shrinkage develops within the first 15 days, after which the values remain nearly constant or increase only slightly. The most important difference between concrete with natural and recycled aggregates was recorded in the case of F15G30 concrete, which has the highest substitution percentage, considering both short and long-term behavior. Specifically, as can be seen in Figure 11, the maximum shrinkage values after 56 days for F15G30 mix were approximately 43% higher with respect to R0, which is related to the highest substitution rate of F15G30. Although all the measured shrinkage values remain within the range typically reported for long-term air-drying shrinkage of conventional concrete (400–800 μm/m),^40^ the increase observed for mixtures with high recycled aggregate contents, particularly F15G30, may limit their suitability for applications requiring high dimensional stability. Excessive shrinkage may promote cracking and negatively affect long-term serviceability, especially in floor slabs, bridge members, and other structural or environmental engineering elements sensitive to shrinkage-induced deformation.

**Figure 10:**
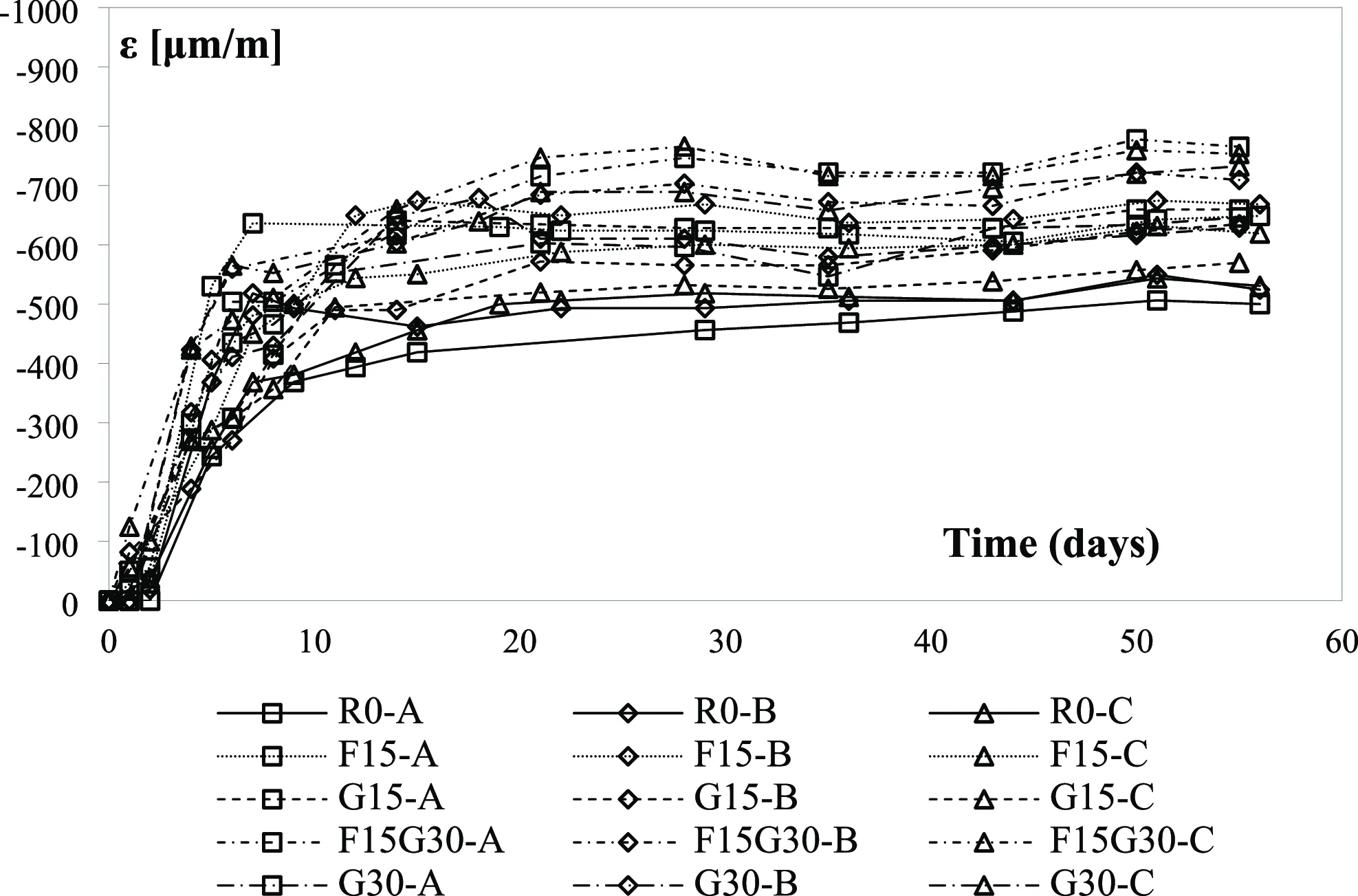
Shrinkage tests results: ε-time curves.

**Figure 11:**
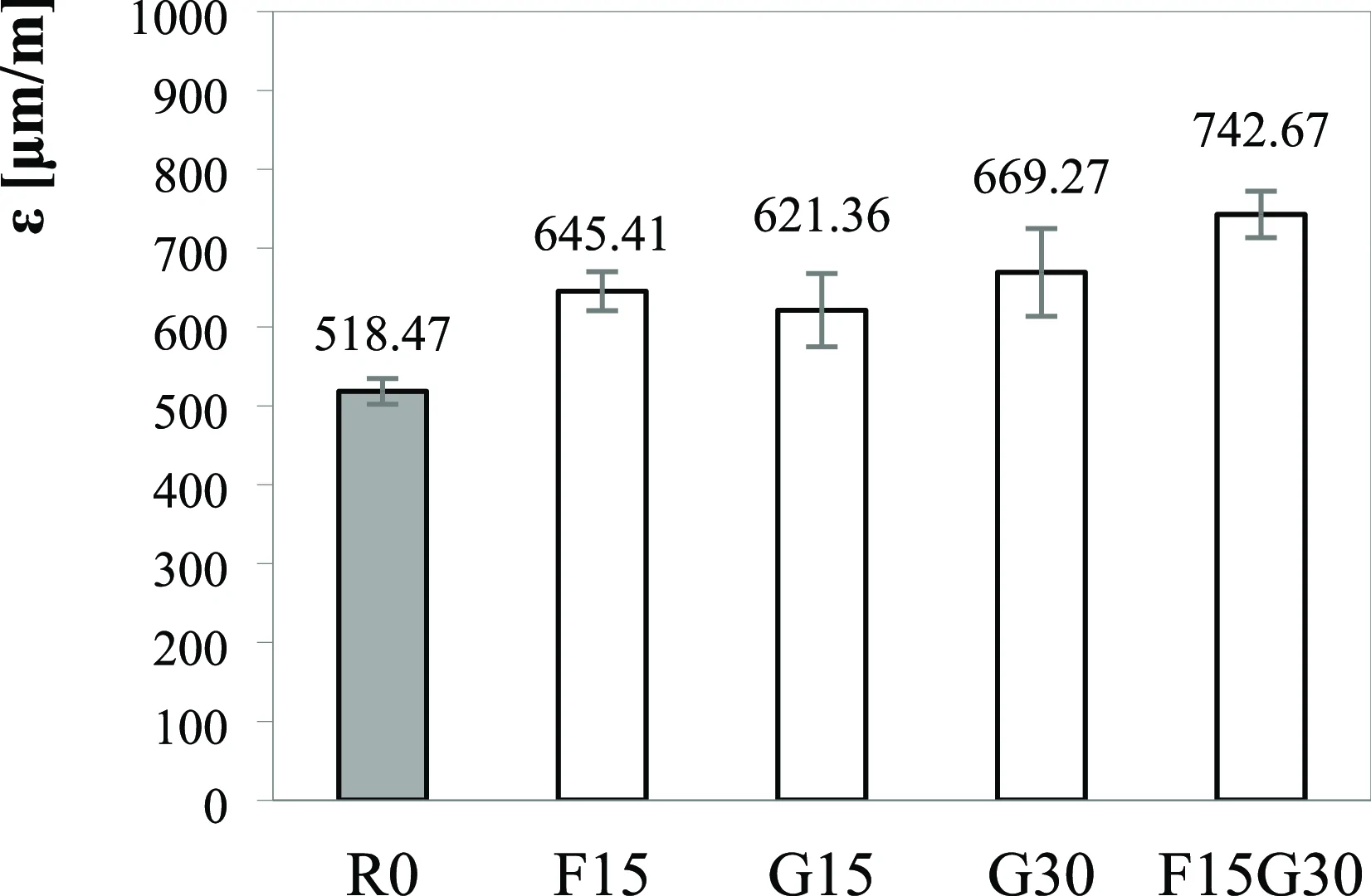
Maximum shrinkage values.

### 4.4 Compressive Strength

As shown in Figure 12, the replacement of natural with composite recycled aggregates has a significant impact on compressive strength. A notable reduction in average compressive strength is observed at 7, 28, and 120 days of curing when CRA are incorporated into the concrete mix, especially for the higher substitution rates.

**Figure 12:**
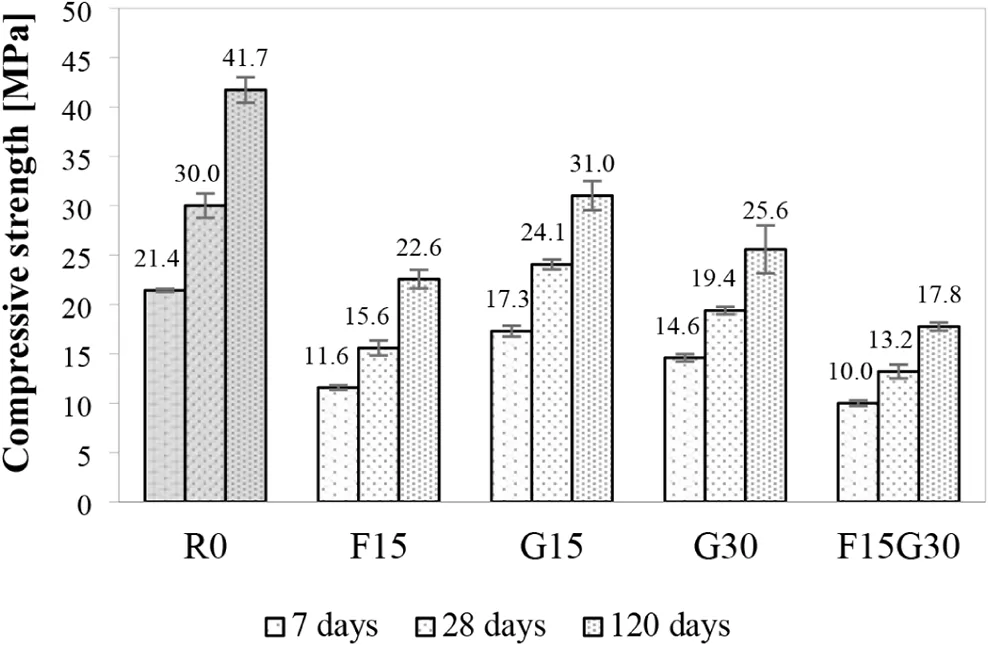
Average compressive strength with the corresponding standard deviation, for 7, 28, and 120 days of curing, for concrete mixes containing composite recycled aggregates (F15, G15, G30 and F15G30), and natural aggregates (R0).

Moreover, the data show that compressive strength decreases with varying severity, not only as the substitution level increases, but also depending on the grain size distribution of the replaced aggregates. The maximum strength reduction is shown in the case of the highest substitution percentage (F15G30 mix), where the strength reduction at 120 days is approximately 57%. Moreover, the reduction tends to be higher when fine aggregates are replaced: the average value measured at 120 days for the G15 specimens containing 15% coarse CRA is approximately 27% higher than that obtained for the F15 specimens containing the same percentage of fine CRA. Moreover, the average compressive strength of the F15 specimens is lower even compared to that of the G30 specimens, despite the latter having twice the substitution percentage.

The same behavior can also be observed at 7 and 28 days. More in detail, the development of compressive strength over time, shown in Figure S1 and Figure 13, indicates that all the mixes containing CRA exhibit a comparable trend with respect to reference NA concrete R0 in terms of rate of increase of strength over time, even if the absolute values are lower.

**Figure 13:**
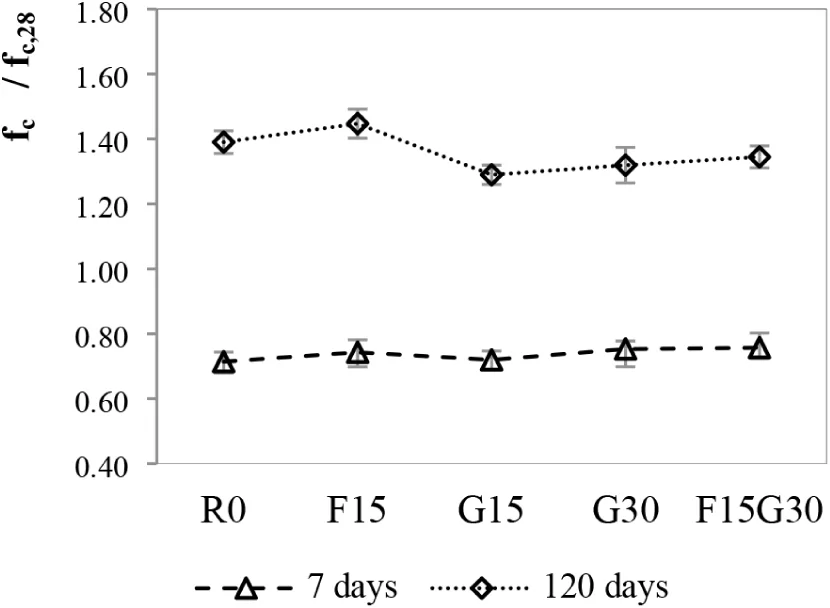
Seven days and 120 days average compressive strength values (f_c_) normalized with respect to the relative 28-day compressive strength (f_c, 28_) with the corresponding standard deviation.

SEM-EDS analyses of the composite recycled aggregates (Figure 3) reveal a highly irregular morphology, along with a heterogeneous composition. When coarse CRA are used (G15), the irregular surface geometry can promote more effective mechanical interlocking with the surrounding cementitious matrix, while the relatively high silicate content provides locally stiffer regions than conventional plastic aggregates, partially mitigating the detrimental effect of the polymeric phase on the mechanical response. Conversely, replacing natural fine aggregates with fine CRA (F15) considerably increases the total interfacial transition zone (ITZ) area. As will be discussed in Section 6, SEM observations of concrete containing CRA reveal localized interfacial voids between the polymeric fraction of the CRA and the cement paste; hence, the larger interfacial area associated with fine particles results in a greater volume of weak ITZ within the concrete. This effect explains the more pronounced reduction in compressive strength observed for the F15 compared with G15, despite the same replacement ratio.

The significant reduction in compressive strength consistently observed at all curing ages, particularly at high replacement levels and for the F15G30 mixture, suggests that these concretes are more suitable for nonstructural applications, especially when fine composite recycled aggregates are used. For structural applications, lower replacement ratios and the exclusive use of coarse composite recycled aggregates would be more appropriate.

### 4.5 Splitting Tensile Strength

The partial replacement of natural with recycled aggregates generally leads to a reduction in splitting tensile strength, as shown in Figure 14a, where the average experimental values after 28 days of curing with the related standard deviations are reported. The only exception is represented by the G15 admixture, which shows a slight increase compared to the reference concrete, amounting to about 7%. This slight improvement may be related to the morphology and size of the coarse CRA. Although the presence of polymeric phases can weaken the ITZ, as discussed for compression strength, the irregular surface morphology of the coarse recycled particles may promote crack deflection and increase the tortuosity of the crack path under tensile loading. In addition, coarse CRA particles may locally contribute to crack-bridging mechanisms before complete debonding, thereby increasing the energy required for crack propagation. These effects can partially compensate for the weaker ITZ and may explain why the G15 mixture exhibited a slightly higher splitting tensile strength than the reference concrete, whereas in the F15 mixture the larger amount of weak ITZ becomes the dominant factor, preventing any improvement with respect to the reference concrete.

**Figure 14:**
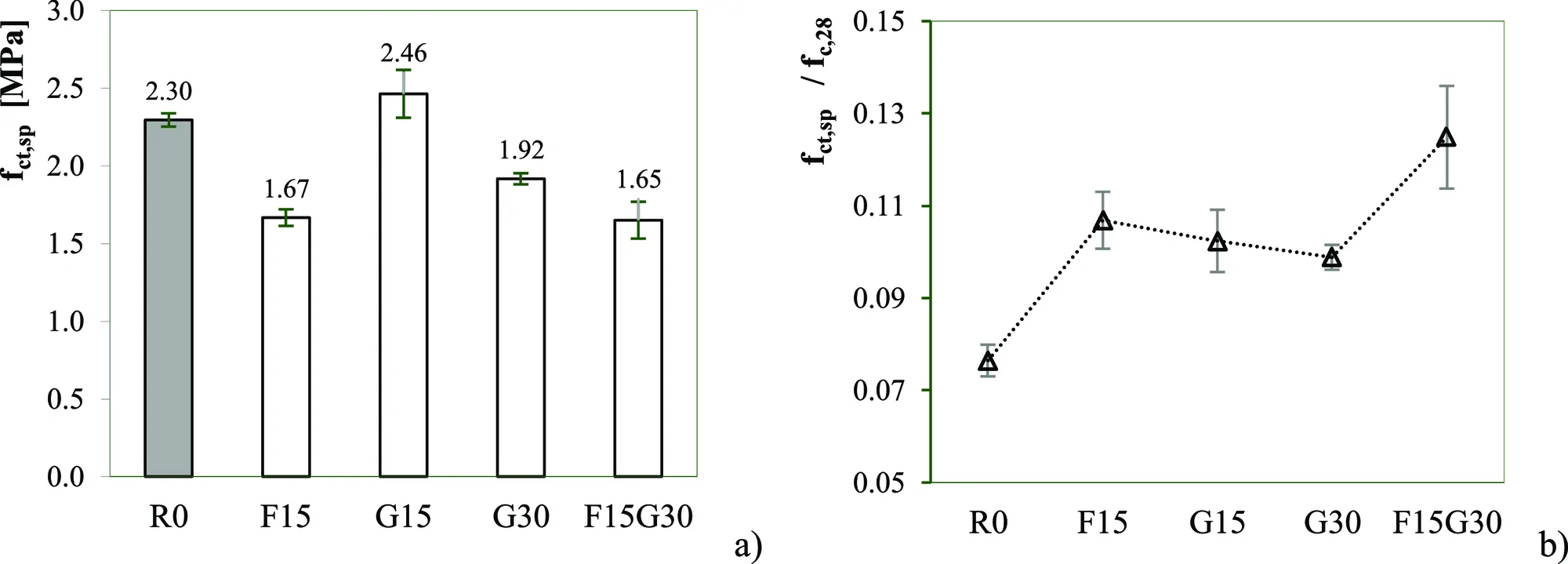
(a) Average 28-day splitting tensile strength with the corresponding standard deviation, for concrete mixes containing composite recycled aggregates (F15, G15, G30 and F15G30), compared to natural aggregate concrete (R0); (b) average 28-day splitting strength normalized with respect to the relative 28-day compressive strength (f_c, 28_) with the corresponding standard deviation.

Anyway, generally speaking, splitting tensile strength appears less negatively affected than compressive strength by the use of composite recycled aggregates: the F15G30 mixture, which exhibited the greatest decrease, has a value that is 28% lower than that of the reference concrete, while the same reduction for compressive strength at the same curing time was about 56%. Figure 14b shows that the ratio between 28-day splitting tensile strength to the corresponding 28-day compressive strength for each mix, is higher for concrete mixes containing CRA, with the F15G30 mixture exhibiting the most significant increase compared to NA concrete. These observations are consistent with the mechanisms responsible for delaying crack initiation and propagation, as further supported by the fracture energy results presented in Section 4.6 and discussed in greater detail in Section 6.

### 4.6 Flexural Strength and Fracture Energy

The results of flexural tests further confirm the observation that composite recycled aggregates exert a less negative impact on the tensile behavior of concrete compared to compressive strength. These results, expressed in terms of peak stress obtained by using eq 1, are presented in Figure 15a. Even in this case, the cementitious mixture with the highest replacement level (F15G30) exhibits the most significant strength reduction, approximately 33%. However, when the replacement involves only 15% of coarse aggregates (G15), the flexural strength remains comparable to that of the reference concrete, showing a reduction of about only 11%. Figure 15b shows that the ratio of 28-day flexural to compressive strength is generally higher for the concretes containing CRA, F15 and F15G30 mixtures exhibiting the most significant increases in comparison to NA concrete. An increasing trend with higher substitution percentages can also be observed for G15 and G30 mixes compared to reference concrete.

**Figure 15:**
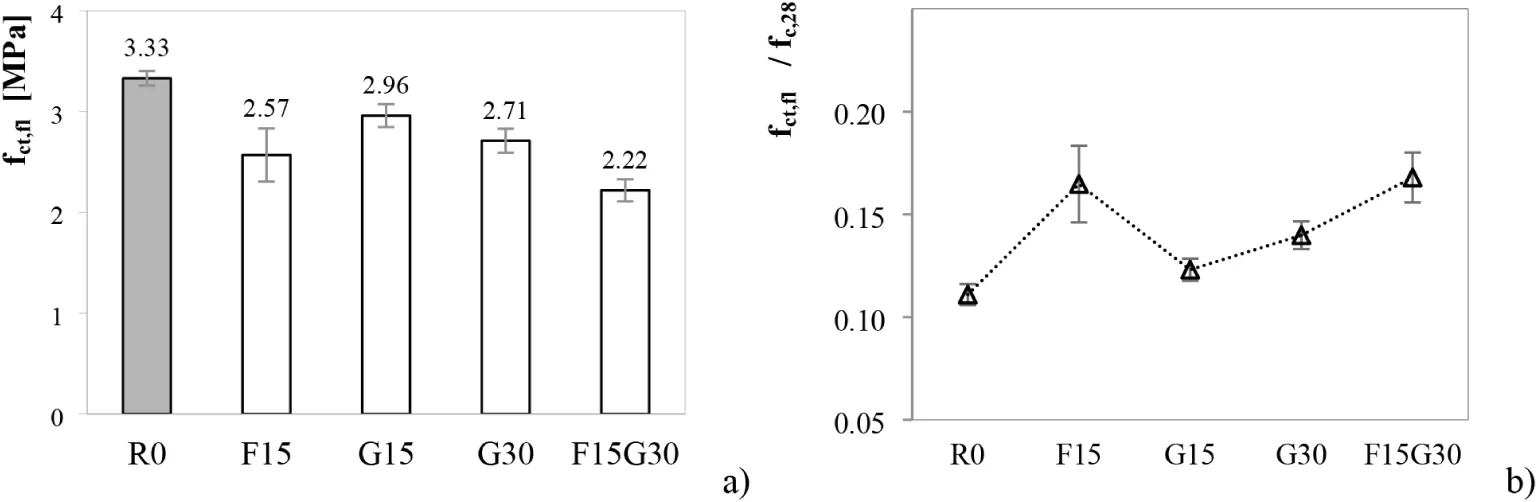
(a) Average 28-day flexural strength, with the corresponding standard deviation, for concrete mixes containing fine (F15), coarse (G15 and G30) and both fine and coarse (F15G30) recycled aggregates, compared to reference concrete (R0); (b) average 28-day flexural strength normalized with respect to the relative 28-day compressive strength (f_c, 28_) with the corresponding standard deviation.

The same tests were also used for the determination of fracture energy, which is calculated by using eq 2, on the basis of the area under P-CMOD curves, representing the amount of energy absorbed until the specimen completely fractures into two parts. Coarse CRA provide an increase in fracture energy values, as shown in Figure 16a, where 9% and 4% higher values can be observed for G15 and G30 compared to NA concrete (R0). On the contrary, the presence of fine CRA, whether as the sole substitute (F15) or in combination with coarse ones (F15G30), leads to a deterioration in the performance of the mixture. In the F15G30 case, however, the negative effect of fine CRA appears to be mitigated by the simultaneous presence of a significant amount of coarse CRA, resulting in a measured fracture energy value comparable (7% lower) to that of the reference NA concrete. Figure 16b shows that the 28-day fracture energy to compressive strength ratio is higher in concretes incorporating CRA, indicating enhanced toughness with respect to compressive behavior. This improvement becomes more evident with increasing substitution levels, as already observed for splitting and flexural strength, and it will be discussed in detail in Section 6.

**Figure 16:**
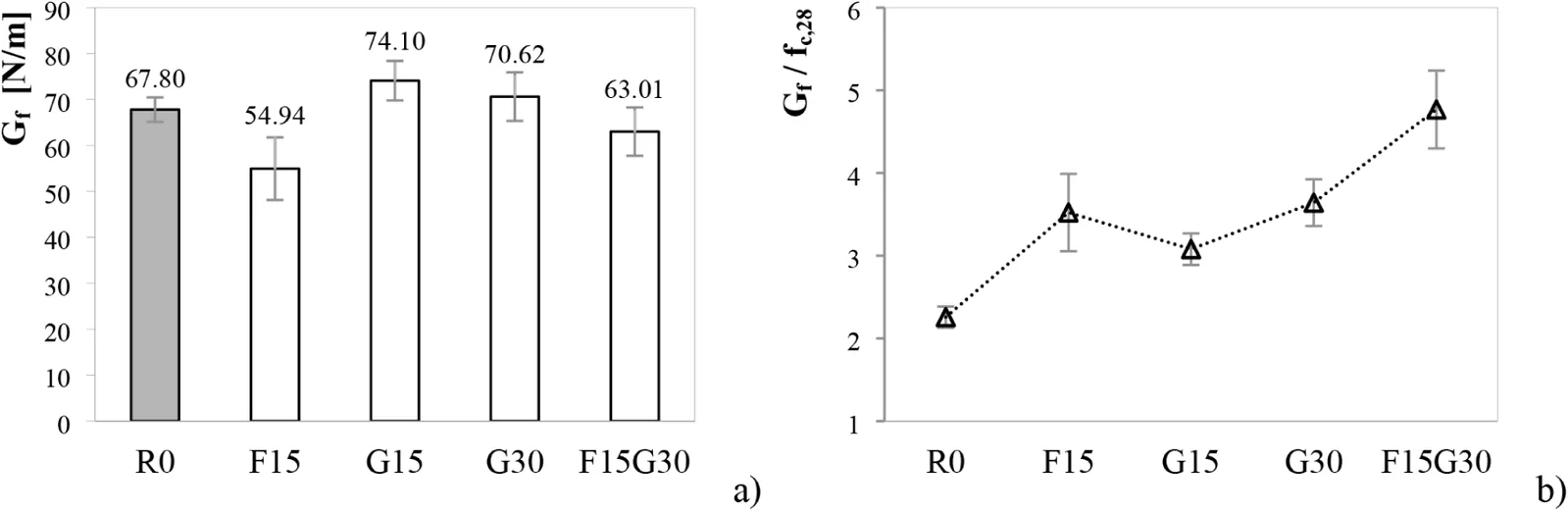
(a) Average 28-day fracture energy, with the corresponding standard deviation, for concrete mixes containing fine (F15), coarse (G15 and G30) and both fine and coarse (F15G30) composite recycled aggregates, compared to reference natural aggregate concrete (R0); (b) average 28-day fracture energy normalized with respect to the relative 28-day compressive strength (f_c, 28_) with the corresponding standard deviation.

### 4.7 Elastic Modulus

The effect of composite recycled aggregates substitution on elastic modulus is shown in Figure 17a: it can be observed that the material stiffness decreases as the replacement percentage increases. In the case of the highest replacement level (F15G30 mixture), the reduction compared to the reference NA concrete R0 is more pronounced, reaching approximately 55%. Similarly to what was observed in the results of the other mechanical tests, for the same replacement percentage, the effect of fine CRA is more significant than that of coarse aggregates: in fact, the G15 admixture exhibits an elastic modulus comparable to that of R0 (with a reduction limited to 12%), whereas for the F15 mixture, this reduction reaches nearly 34%. This behavior can be attributed to the same mechanisms discussed for compressive strength. The use of coarse CRA limits the total ITZ area while the irregular morphology of the recycled particles promotes a more effective mechanical interlocking with the surrounding cement paste. Conversely, replacing natural fine aggregates with fine CRA considerably increases the total ITZ area, where localized interfacial voids are more likely to develop because of the polymeric fraction of the composite aggregate. As a result, the higher volume of weak interfaces leads to a more pronounced reduction in stiffness, explaining the better performance of the G15 mixture.

**Figure 17:**
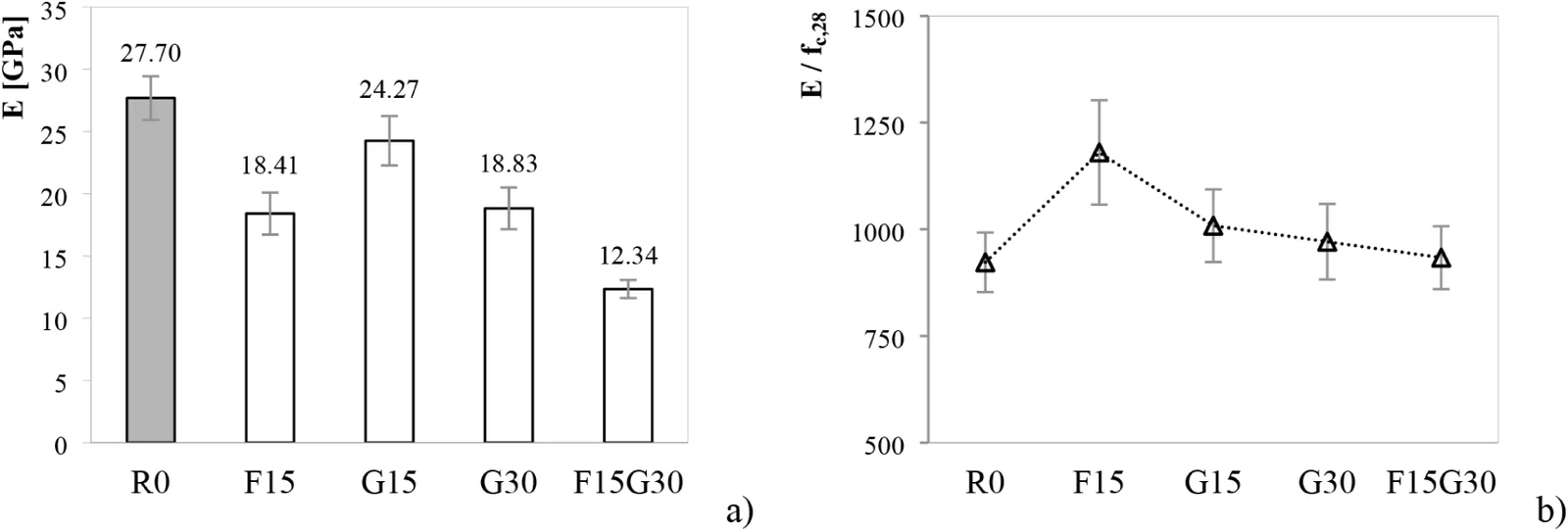
(a) Average 28-day elastic modulus, with the corresponding standard deviation, for concrete mixes containing fine (F15), coarse (G15 and G30) and both fine and coarse (F15G30) recycled aggregates, compared to reference concrete (R0); (b) average 28-day elastic modulus normalized with respect to the relative 28-day compressive strength (f_c, 28_) with the corresponding standard deviation.

Furthermore, Figure 17b shows the ratio between the 28-day elastic modulus and the related compressive strength for each mix, which remains approximately constant for all the mixtures, indicating that the reduction in stiffness follows the same mechanisms governing the loss of compressive strength.

Accordingly, the same considerations regarding the practical applications of concretes containing composite recycled aggregates, discussed for compressive strength, also apply to the elastic modulus. The marked reduction in stiffness observed with increasing replacement levels, especially when fine CRA are used, further supports the use of these concretes only for nonstructural applications, particularly for mixes incorporating fine fractions and/or high replacement levels, such as F15G30.

## 5 Environmental Performance

The environmental implications of replacing fine and coarse natural aggregates with composite recycled aggregates were also investigated. Indeed, the implementation of circularity strategies does not always correspond to an actual reduction in environmental impact. This is because the use of recycled materials within an open-loop system, where the material is assigned an application different from that of its previous life cycle, often entails additional processing steps that may outweigh the environmental savings associated with avoiding the extraction of new resources.

The first focus of the environmental analysis therefore concerned the recycling process of composite material discarded from sink manufacturing processes, with the aim of producing coarse and fine recycled aggregates. The focus subsequently shifted to the comparison of the different concrete mixes. Environmental impacts were quantified using the Life Cycle Assessment (LCA) methodology, as defined by the EN ISO 14040 and 14044 standards, according to which an LCA study is structured into four iterative phases: Goal and scope definition, Life Cycle Inventory (LCI), Life Cycle Impact Assessment (LCIA), and Interpretation of results. In addition to these general rules applicable to all systems, the Product Category Rules (PCR) for construction products were adopted, specifically the Construction Products PCR 2019:14, version 1.11. These provide specific guidance on the functional unit (FU) to be used for comparing the different mixes, the life cycle stages to be included in the analysis (physical system boundaries), the exclusion criteria for certain flows, allocation procedures, the approach to inventory data modeling, and the selection of the impact assessment method. The analysis was carried out with the SimaPro software, version 10.1, with the Ecoinvent database 3.10.

### 5.1 Goal and Scope Definition

At this stage, the goal of the study was first defined, namely to verify whether the partial replacement of fine and coarse aggregates with composite recycled aggregates leads to a reduction in the environmental impacts of the F15, G15, G30, and F15G30 mixes compared with the reference concrete R0. The comparison was carried out on 1 m^3^ of concrete, which represents the FU, the quantity to which all inventory data and impact results are referred. The analysis covers up to concrete production according to a “cradle-to-gate” approach, as shown in Figure S2. For each concrete mix the life cycle stages considered range from raw material extraction to component manufacturing. The final mixing phase was not included, as it is identical for all mixes and therefore not relevant for a comparative study aimed at identifying potential impact reductions attributable to the implemented circularity strategy. With regard to geographical boundaries, the analysis was contextualized within the Italian area, and this aspect was taken into account during system modeling in the Life Cycle Inventory (LCI) phase.

### 5.2 Life Cycle Inventory (LCI)

The second phase of the LCA involves the collection of consumption data associated with each stage included within the system boundaries. System modeling in the SimaPro software was carried out by linking the consumption flows to appropriate Ecoinvent data sets, which provide emission data to air, water, and soil. The modeling was performed according to the cutoff approach implemented in the selected Ecoinvent data sets used to represent the different concrete components. As the specific origin of each component was not known, data sets belonging to the “market activities” category were adopted; these include average transport processes representative of the geographical area to which each data set refers. In most cases, the geographical scope of the data sets is broader than the Italian context. Details of the component modeling are reported in Table 4.

**Table 4:** Modelling of the Virgin Components of the Concrete Mix

**Component**	**Ecoinvent data set**
Cement	*Cement, Portland {Europe without Switzerland}| market for cement, Portland | Cut-off, S*
Water	*Tap water {Europe without Switzerland}| market for tap water | Cut-off, S*
Fine NA	*Sand {CH}| market for sand | Cut-off, S*
Coarse NA	*Gravel, crushed {CH}| market for gravel, crushed | Cut-off, S*
Superplasticizer	*Polycarboxylates, 40% active substance {RER}| market for polycarboxylates, 40% active substance | Cut-off, S*

With regard to the modeling of recycled aggregates, they are obtained exclusively through the milling of the waste material. Collection, storage, sorting, transportation, and material losses were not included in the system boundaries, as the present study is based on laboratory-scale experiments in which the waste material was directly available for processing and characterization. Depending on the required degree of fineness, the energy consumption of the mill used for the milling process varies. The selected industrial plant features the following installed power ratings for its main components: 18.5 kW for the air compressor, 37 kW for the crushing system, and 192 kW for the ball mill. The operation of the ball mill allows the system to reduce the material to particles with a size of 15 μm, significantly below the particle size of both coarse and fine CRA; hence, the production of coarse aggregates requires only the crusher. Consequently, the electricity consumption for the production of 1 kg of granulate amounts to 0.021 kWh and 0.035 kWh for coarse and fine CRA, respectively. In assessing the environmental impact of electricity consumption, it is essential to consider the energy source. Depending on whether electricity is drawn from the national grid or generated from renewable sources, the resulting impact differs. Given that the design of new production systems should account for both circularity and the use of renewable resources, a scenario in which the electricity used is generated from photovoltaic renewable sources was analyzed (*Electricity, low voltage {IT} | electricity production, photovoltaic,* 3 *kWp slanted-roof installation, multi-Si, panel, mounted | Cut-off, S*). From the environmental burdens generated by the milling process, the avoided impact associated with the prevention of landfill disposal must be subtracted. In the absence of a representative Ecoinvent data set for composite waste, a separate modeling of the degradation of the inert fraction (*Inert waste {RER} | treatment of inert waste, sanitary landfill | Cut-off, S*) and of the plastic fraction (*Waste plastic, mixture {CH} | treatment of waste plastic, mixture, sanitary landfill | Cut-off, S*) was performed. The distinction between the two flows does not imply any actual separation of the composite material components, but serves to represent the different landfill behavior of the materials constituting the waste.

### 5.3 Life Cycle Impact Assessment (LCIA)

For the impact quantification, the EN 15804 method,^43^ which includes 13 impact indicators, was applied. Ecotoxicity and human toxicity indicators were excluded, and the analysis focused on the following environmental indicators: Climate Change (kg CO_2_ eq), Acidification (mol H^+^ eq), Marine Eutrophication (kg N eq), Freshwater Eutrophication (kg P eq), Terrestrial Eutrophication (mol N eq), Land Use (Pt), Ozone Depletion (kg CFC-11 equiv), Photochemical Ozone Formation (kg NMVOC eq), Fossil Resource Use (MJ), Mineral and Metal Resource Use (kg Sb eq), and Water Use (m^3^ deprived). Each indicator is characterized by an equivalent unit of measure that aggregates the contributions of the various emission flows associated with the specific environmental issue. In this way, the inventory analysis results are converted into impact indicators.

A preliminary impact assessment was first performed on CRA considered individually, in order to determine whether the avoided impact associated with the prevention of landfill disposal is sufficient to offset the additional impact of the milling treatment. The results obtained, referred to 1 kg of fine and coarse CRA, are reported in Table S1 and graphically represented in Figure 18 for coarse CRA. It can be observed that, for both coarse and fine CRA, in the majority of impact categories the contribution of the avoided impact exceeds that of the milling process, as indicated by the resulting negative impact values. Clearly, negative impact results do not represent an intrinsically negative environmental footprint of recycled aggregates, but they have a purely mathematical meaning and can be interpreted as environmental credits arising from the balance between burdens and benefits. It can also be noted that only two impact categories, namely *Resource Use, minerals and metal*s, and *Water Use*, exhibit values greater than zero. By analyzing the contributions of the milling process and the avoided impact associated with landfill disposal, reported in Table S2 for 1 kg of coarse CRA, it can be observed that in the *Resource Use, minerals and metals* category the avoided impact contributes to reducing the overall impact, but not to an extent sufficient to generate environmental credits. With respect to the Water Use category, it can be noted that the impact associated with landfill disposal has a negative sign, indicating that landfilling is a system capable of returning water to the environment, due to meteoric water infiltration and leachate. Avoiding landfill disposal therefore implies avoiding this return of water to the environment; as a result, the Water Use associated with coarse CRA is greater than zero and exceeds the impact associated with the milling process alone.

**Figure 18:**
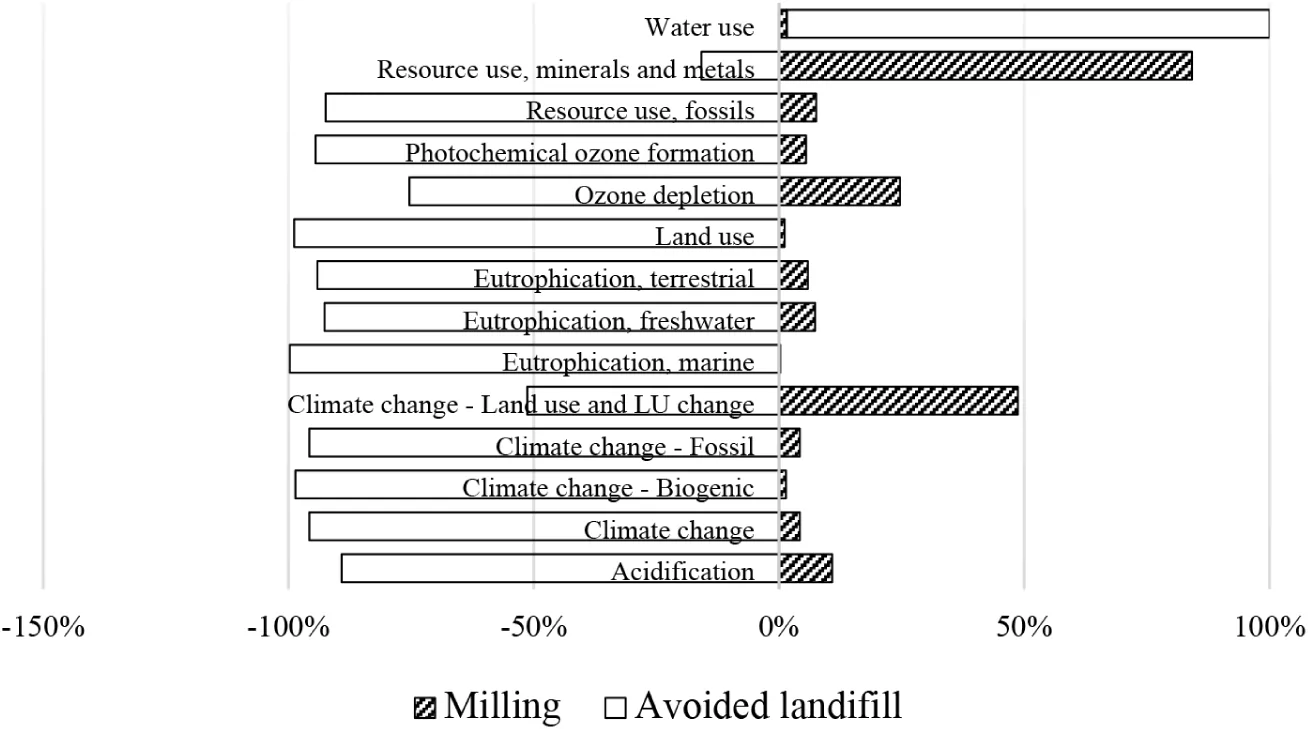
Percentage contribution of the milling process and avoided landfill to the overall impact of coarse CRA.

### 5.4 Analysis of the Results

Once the potential environmental benefit achievable through the use of CRA in the mix had been verified, the environmental impacts for 1 m^3^ of each concrete mix were quantified and compared, as reported in Table S3. From the graphical representation in Figure 19, it can be observed that, as the replacement percentage increases, environmental impacts decrease, with the exception of the Water Use category due to the effects of the avoided impact discussed in Section 5.3. Therefore, the mix F15G30 shows the overall best environmental performance. The greatest environmental benefits are achieved in the Marine Eutrophication category, where the F15G30 mix exhibits a negative impact result, and in the Land Use category, where the difference between R0 and F15G30 amounts to 73%. For Marine Eutrophication, the substantial environmental benefit associated with the avoided impact is linked to the prevention of nitrogen compound emissions from landfill leachate, whereas for Land Use it is related to the long-term occupation of land by landfill, which leads to ecosystem alterations.

**Figure 19:**
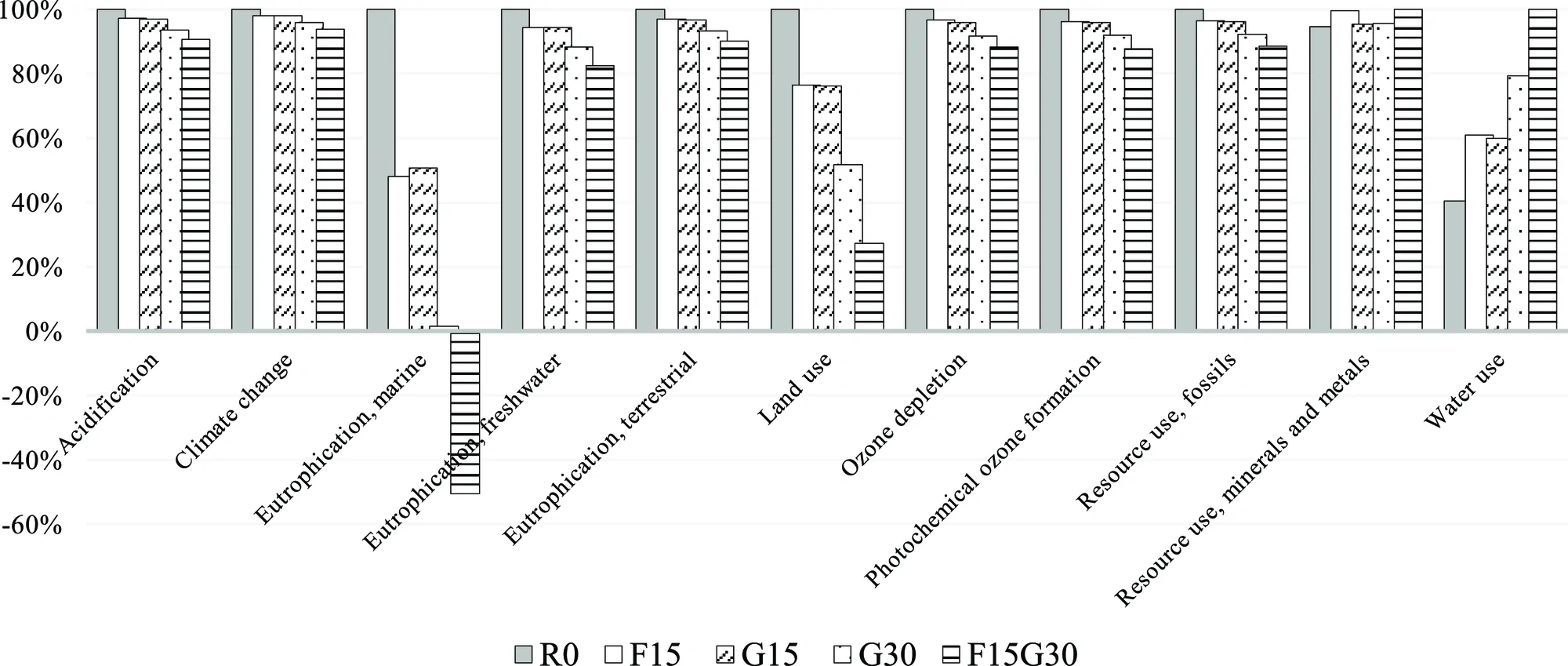
Comparative percentage results referred to 1 m^3^ of each concrete mix.

### 5.5 Sensitivity Analysis

In order to assess the robustness of the environmental results with respect to the modeling assumptions adopted for both the natural aggregates being replaced and the recycled aggregates used as replacement materials, a sensitivity analysis was performed. Regarding natural aggregates, alternative data sets representative of an extra-European geographical context (RoW) were adopted for both fine and coarse natural aggregates. Since no data sets specifically representative of the Italian or broader European context were available, the baseline assessment relied on Swiss (CH) data sets. The percentage deviation between the environmental impacts of the fine and coarse aggregate data sets associated with the two geographical contexts was found to be significant across all impact categories, with average variations generally exceeding 50%. Figure 20a reports the results for fine aggregates, while a comparable trend was observed for coarse aggregates. However, the variation is expected to be considerably less relevant when assessing the impacts of the concrete mixtures, since natural aggregates are not the main drivers of the overall environmental impacts. This assumption is confirmed by the radar chart shown in Figure 20b which reports the results for the F15G30 concrete mixture as an illustrative example. When alternative geographical data sets are adopted for fine and coarse natural aggregates, the percentage variation in the environmental impacts of the concrete mixture is substantially reduced. For the Climate Change impact category, for instance, the variation is limited to only 3%. It should also be noted that, when comparing the different concrete mixtures, including the reference concrete, the consistent application of an alternative modeling approach for natural aggregates across all mixtures does not affect the environmental ranking of the investigated solutions.

**Figure 20:**
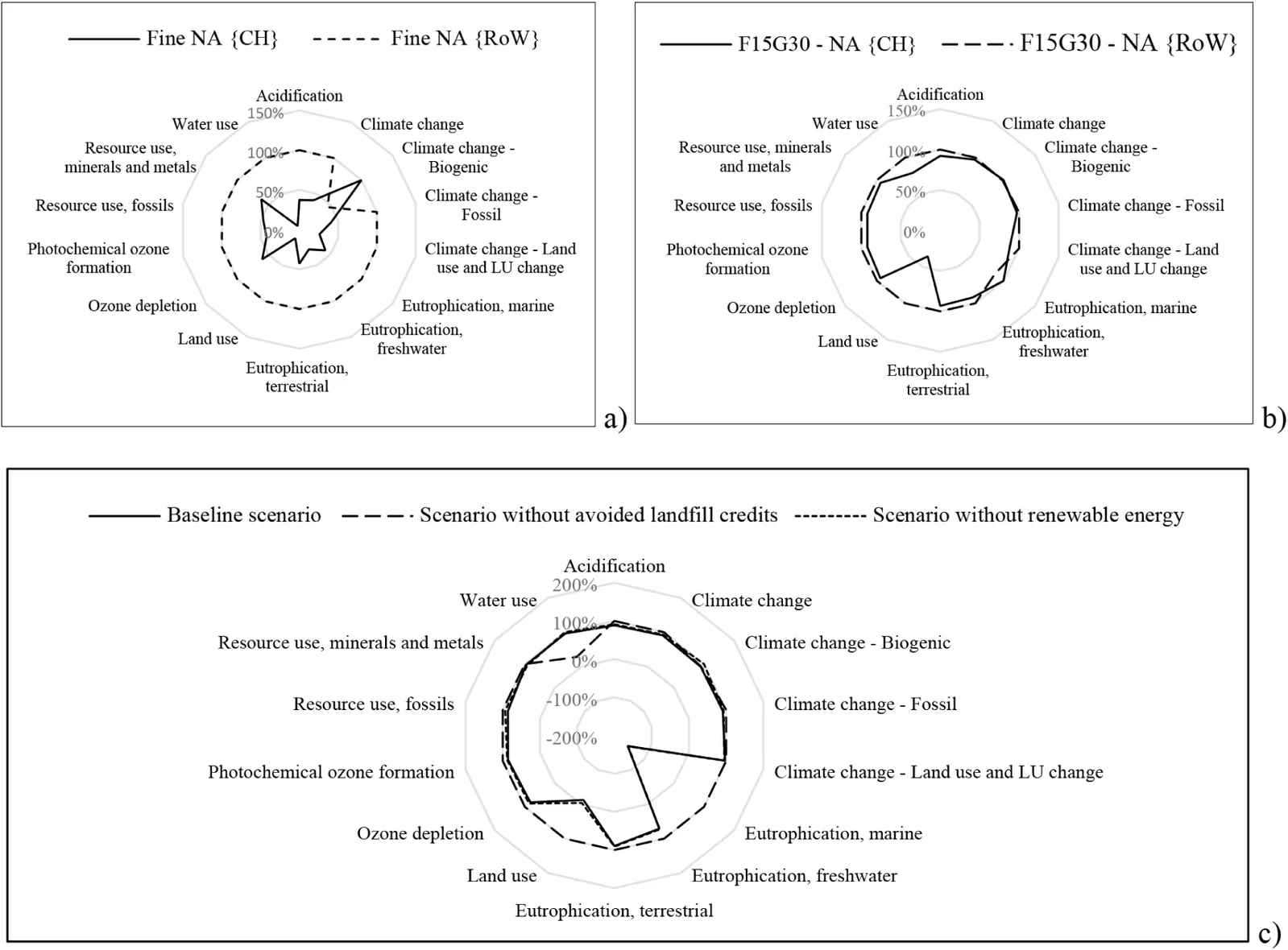
(a) Radar diagram showing the percentage variation in the environmental impacts of fine natural aggregates obtained using data sets from different geographical contexts; (b) sensitivity analysis on 1 m^3^ of F15G30 concrete using alternative geographical data sets for fine and coarse natural aggregates; (c) sensitivity analysis of recycled aggregate modeling and electricity supply scenario for F15G30 concrete mix.

A much more influential parameter affecting the environmental ranking of the investigated concrete mixtures is the modeling approach adopted for recycled aggregates. In the baseline scenario, the environmental benefit associated with recycled aggregates derives from the avoided landfill treatment of the composite waste. This approach is methodologically acceptable when the composite material discarded from sink manufacturing is effectively classified as waste. Under this assumption, the recycling scenario receives the environmental credits associated with the avoidance of landfill disposal. Conversely, if the discarded composite material is not classified as waste, system expansion cannot be applied and no avoided landfill credit can be assigned to the recycled aggregates. In this alternative scenario, recycled aggregates only incorporate the environmental burdens associated with the milling process, which are no longer offset by any additional environmental credit. Keeping the modeling approach based on avoided landfill credits unchanged, an additional parameter that can significantly influence the environmental performance of recycled aggregates is the electricity source adopted for the milling process. In the baseline scenario, electricity generated from photovoltaic renewable sources was assumed. This choice was motivated by the fact that the development of circular production systems is often associated with the adoption of renewable energy sources in order to maximize the potential environmental benefits of recycling strategies. Nevertheless, the environmental performance of recycled aggregates may differ when electricity is supplied from the national grid. Therefore, an additional sensitivity analysis was performed by replacing the photovoltaic electricity data set with the Italian low-voltage electricity market mix (*Electricity, low voltage {IT} | market for electricity, low voltage | Cutoff, S*). Under this scenario, a reduction in the environmental benefits associated with the replacement of natural aggregates is expected due to the higher impacts associated with grid electricity production. To evaluate the magnitude of this effect, the analysis focused on the F15G30 mixture, which exhibited the greatest environmental improvement in the baseline assessment. The variation in environmental results associated with both the alternative modeling approach for recycled aggregates (without avoided landfill credits) and the alternative electricity supply scenario is presented in Figure 20c. The two alternative scenarios are identified according to the modeling assumption modified with respect to the baseline scenario, namely “without avoided landfill credit” and “without renewable energy”. The results show that the “without avoided landfill credit” scenario is associated with the highest environmental impacts across most impact categories, although, for the environmental indicators that are not substantially influenced by the avoided landfill credit, the results remain robust, with limited percentage differences between the impacts obtained under the different scenarios (a maximum variation of 7 percentage points for the Climate Change indicator). Conversely, the results obtained for Marine Eutrophication, Land Use, and Water Use are more sensitive to the modeling assumptions adopted, showing substantially larger variations.

## 6 Discussion

Based on the results presented in the preceding sections, the effects of composite recycled aggregate (CRA) incorporation are discussed by integrating the observed physical, durability, and mechanical performance with the outcomes of the life cycle assessment (LCA). The discussion focuses on three main aspects: (i) the microstructural mechanisms governing the changes induced by CRA replacement, with particular attention to particle size, interfacial transition zone characteristics, and crack propagation; (ii) the extent to which the behavior of CRA concrete differs from or resembles that reported for concrete incorporating conventional recycled plastic aggregates; and (iii) the balance between mechanical performance and environmental benefits, considering how increasing CRA replacement levels affect both material properties and the main LCA indicators. This framework is intended to provide a comprehensive interpretation of the experimental results and to identify the conditions under which CRA incorporation may represent a technically and environmentally viable strategy for concrete production.

### 6.1 Microstructural Mechanisms Governing Physical, Durability, and Mechanical Performance

The study demonstrates that the use of composite recycled aggregates has a significant impact on the physical and mechanical properties of concrete, with changes that depend not only on the replacement percentage of natural aggregates but also on the particle size of the waste materials, as can be seen in Figure 21.

**Figure 21:**
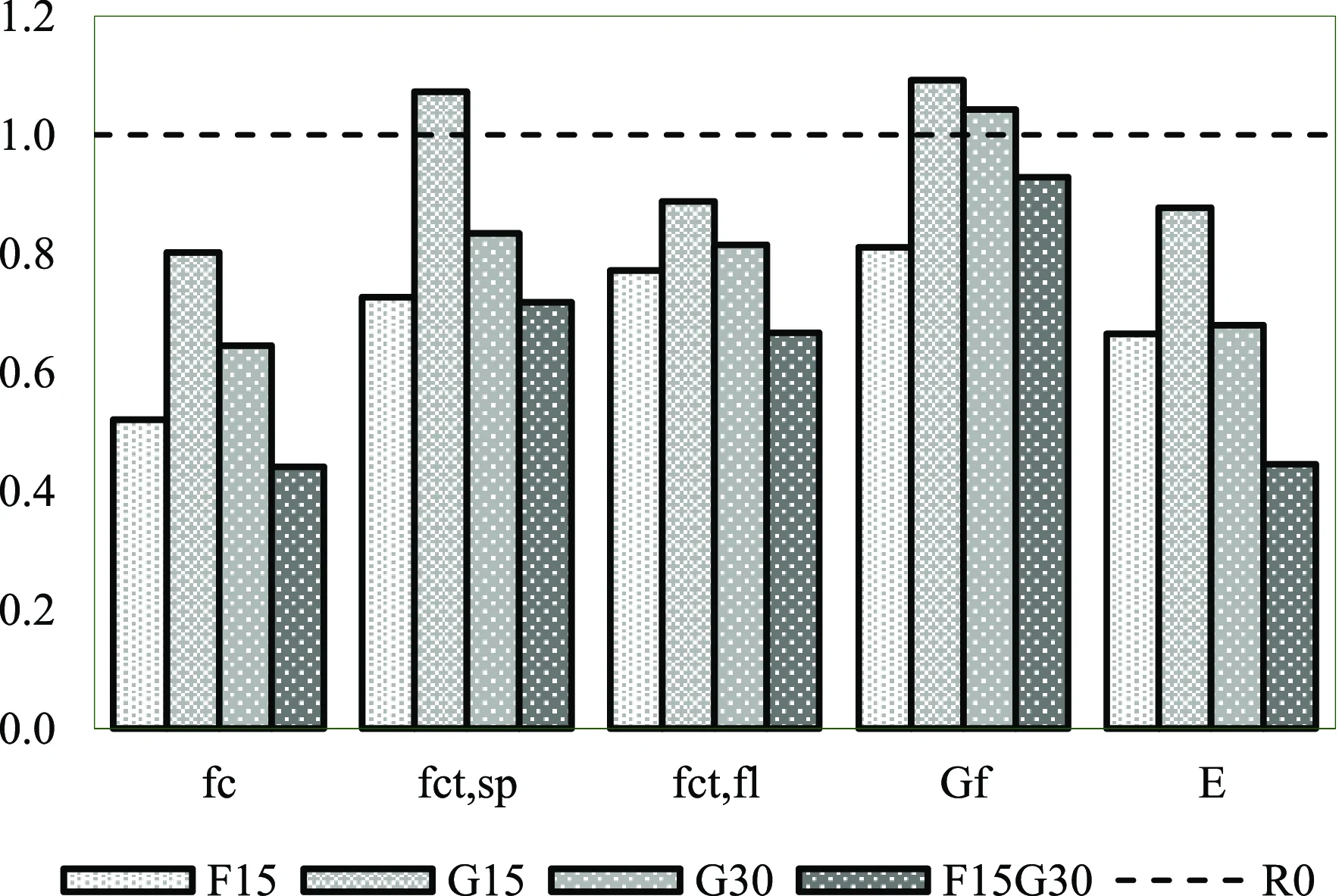
Comparison of the normalized mechanical properties of F15, G15, G30, and F15G30 mixtures with respect to the reference mixture R0 (dashed line, normalized value equal to 1): compressive strength f_c_, splitting tensile strength f_ct, sp_, flexural tensile strength f_ct, fl_, fracture energy G_f_, and elastic modulus E for 28 days of curing.

First, a decrease in both fresh and hardened density was observed for all the mixes containing CRA, due to their lower particle density in comparison to NA. However, the reduction is more pronounced when fine CRA are introduced into the concrete, either alone or in combination with coarse ones, due to the increased porosity of these mixes, which is associated with the larger specific surface area of fine recycled aggregates and the consequent development of a greater volume of weak ITZs between the recycled particles and the cementitious matrix. In this context, particle size represents a key governing parameter, resulting in more pronounced reductions not only in terms of density, but also when analyzing compressive strength and stiffness, as can be seen in Figure 21.

From a microstructural perspective, despite the relatively high quartz content in the composite material composition, the presence of plastic compounds results in a degradation of the physical and mechanical properties of concrete, due to weaker interfacial transition zone (ITZ) between the recycled aggregates and the cement paste. As a matter of fact, the hydrophobic nature of the plastic components and the substantially hydrophilic behavior of the cement matrix leads to weak adhesion between the two materials (wall effect), with the consequent formation of an empty interface and an increase in the porosity of the concrete.

This interface mechanism leads to a strong reduction of compressive strength, as can be seen in Figure 21, especially in case of fine CRA and high replacement. More in detail, the chemical incompatibility between the thermoset fraction contained in CRA and the cement matrix causes the presence of nonabsorbed water, which results in reduced cement hydration reaction near the surface of the aggregates and the aforementioned empty interface, worsening the ITZ between the two materials, as clearly visible also by SEM images in Figure 22b and Figure 22c; in contrast, the interface in the R0 sample between the traditional aggregates and the cement paste shows no discontinuities, as can be seen in Figure 22a.

**Figure 22:**
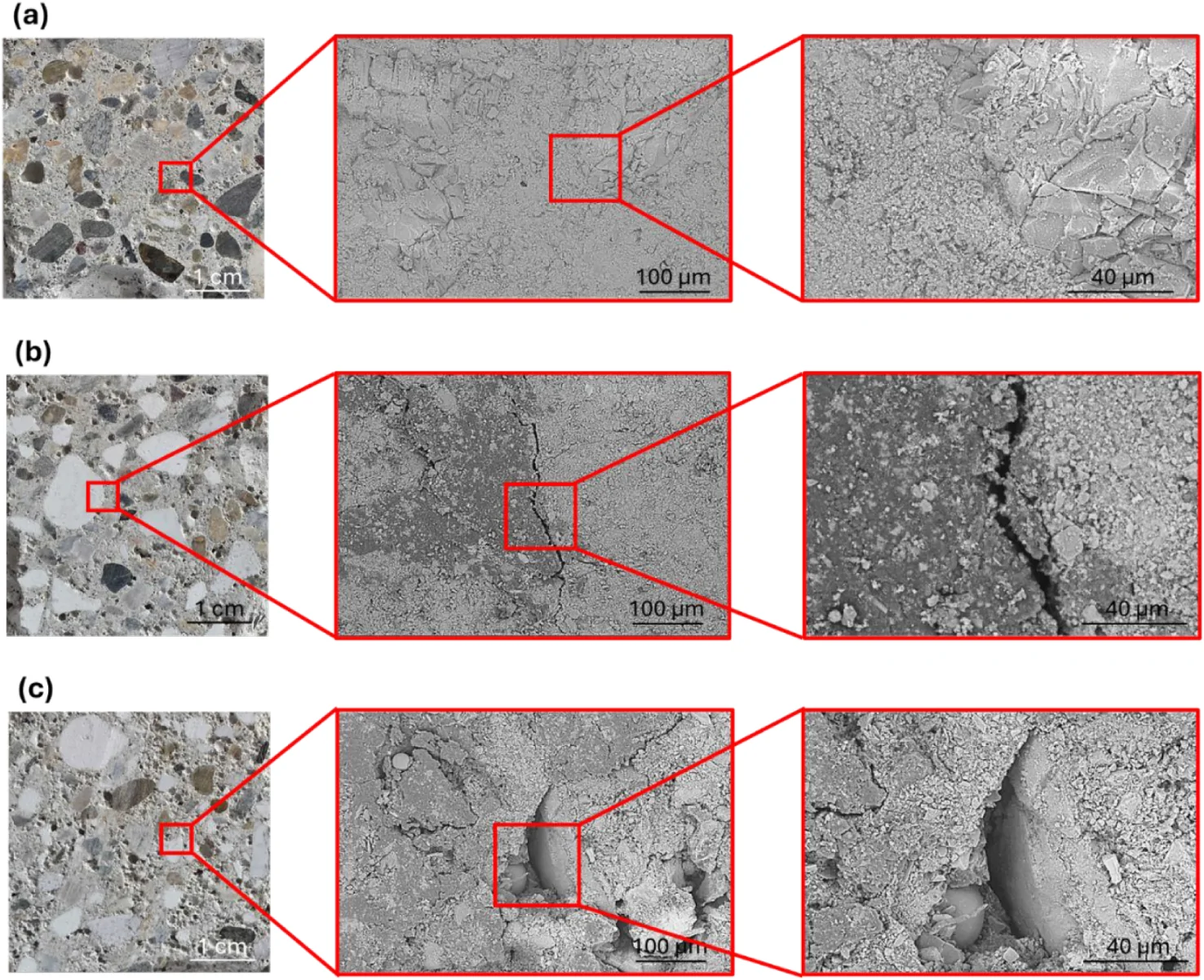
Optical microscope image of R0 sample (a), with the related SEM images focusing on concrete-aggregate interface; optical images of G30 (b) and F15G30 (c) samples, with SEM enlargement of the cement-waste interface.

Figure 22 shows representative SEM micrographs taken of concrete sections at various magnifications, illustrating the interface between the composite recycled aggregates and the surrounding cementitious matrix. The observations were repeated in several independent areas of the specimens, and the images presented are representative of the overall microstructure. The images in Figure 22b and Figure 22c show, respectively, a cross-section of the G30 and F15G30 samples and the related enlargements at the interphase between the CRA and cement. The recycled aggregates can be distinguished from the surrounding hydrated cement paste by their characteristic morphology, consistent with that observed before concrete production (Figure 3), and by their darker contrast in the SEM images, resulting from the higher carbon content of the polymeric matrix, which generates less elastic electron scattering compared to the concrete matrix. At the interface, a separation between the cement matrix and the organic base of the aggregate can be noted, indicating an incomplete compatibility and wettability of the two phases. Accordingly, the interfacial transition zone appears generally more discontinuous in concretes containing CRA than in reference concrete, which exhibits a more continuous contact between the aggregate and the cement matrix. In particular, for the sample F15G30, some spherical structures that were part of the CRA are still visible and have not bonded to the concrete. More in detail, in specimens with natural aggregates, the cracked surface was predominantly located within the cement paste, suggesting that the deformation between the components occurs in a more cohesive manner under loading, contributing to good mechanical performance. Conversely, in specimens incorporating CRA, the failure surface shows significant propagation along the interface between the cement matrix and the waste material, suggesting a reduced interfacial adhesion between the two materials, resulting in reduced compressive strength.

The weak ITZ between the composite recycled aggregate surface and the cement matrix, while negative for compressive strength, tends to hinder crack propagation, thereby resulting in less pronounced adverse effects on tensile behavior and fracture energy, as can be seen in Figure 21. The presence of discontinuities along the crack path promotes mechanisms such as crack deflection and deviation around the recycled aggregates. Additionally, due to their lower modulus of elasticity, composite recycled aggregates partially absorb the internal stress, promoting a redistribution of the load within the matrix. These effects delay crack initiation and propagation, allowing the material to absorb more energy before failure occurs, and thus enhancing the overall energy dissipation capacity of the concrete.

It can be stated that the overall performance of the investigated concretes is governed by the balance between two competing microstructural mechanisms: the detrimental effect of the polymeric phase, which weakens the interfacial transition zone, and the beneficial contribution of the mineral quartz fraction, together with the irregular morphology of the recycled aggregates, which promote mechanical interlocking and energy dissipation, particularly when coarse fractions are used. As a result, although both CRA and recycled plastic aggregates exhibit reductions in strength-related properties due to weak interfacial bonding, the mineral-rich composition of CRA substantially modifies the overall mechanical response by limiting strength losses (especially for tensile loading) and enhancing fracture resistance. Consequently, concretes incorporating CRA exhibit a mechanical behavior that differs in several aspects from that commonly reported for fully polymeric recycled aggregates, highlighting the importance of considering the hybrid nature of this waste material when assessing its suitability for concrete production.

Overall, the trends in the mechanical and physical properties of the various mixtures, shown in Figure 21, indicate that fine-grained CRA primarily causes a loss of stiffness and strength, while coarse-grained CRA leads to an increase in toughness; these two effects roughly offset each other in mixed-size aggregates such as F15G30. This decoupling between compressive behavior and tensile/fracture behavior is not typical for concretes containing conventional plastic aggregates, for which the literature generally reports a more uniform degradation across all mechanical properties. This indicates that particle size distribution, rather than the overall replacement ratio alone, is the key factor in tailoring CRA concrete to a specific end use, with a low-to-moderate replacement of coarse aggregates favoring structural applications, while mixtures rich in fine aggregates are better suited for nonstructural applications.

### 6.2 Comparison with Conventional Recycled Plastic Aggregates

As regards both fresh and hardened density, reductions comparable to those observed in the present study have been consistently reported in the literature with increasing levels of aggregate replacement in concretes incorporating recycled plastic aggregates, mainly due to the lower particle density of the polymeric fraction compared to natural aggregates.^6,7,18,44^ However, the density reduction observed in the present study remains relatively limited despite the hybrid nature of CRA, suggesting that the high quartz content partially compensates for the low density of the polymeric phase. Moreover, the greater density reduction observed when fine CRA are used can be attributed to their larger specific surface area and to the partial incompatibility between the cement matrix and the composite recycled aggregates, a microstructural mechanism that has already been reported in the literature for concrete incorporating plastic waste aggregates.^6,45,46^

Consequently, the higher water absorption values, in terms of total absorption and penetration under pressure, for CRA concrete compared to reference NA concrete are consistent with the increased porosity. Increased water absorption has been widely reported for concretes containing recycled plastic aggregates;^6,44,47^ however, the relatively limited increase observed in the present study indicates that the quartz fraction mitigates the detrimental effect of the polymeric component, allowing the concrete to maintain durability-related properties within the range generally accepted for structural applications. This is also confirmed by the comparable sorptivity values observed across all the tested mixes, suggesting generally adequate durability performance, even under potentially aggressive exposure conditions.

The results related to drying shrinkage are consistent with the literature on concretes containing plastic aggregates in replacement of natural ones.^6,48^ Most of the studies report higher drying shrinkage compared to conventional concrete, further highlighting a tendency to increase as the substitution level increases. The reason for this can be attributed, on one hand, to the lower stiffness of aggregates containing plastic, as a result of which, they provide lower resistance to the shrinkage of cement paste compared to natural aggregates.^48–50^ On the other hand, the presence of voids at the interfacial transition zone (ITZ) between composite recycled aggregates and the cement matrix also contributes to the increased shrinkage observed in CRA concrete. When the specimens are subjected to air curing, the evaporation of the water contained in the pores—more abundant in concretes with CRA due to their higher overall porosity—leads to volumetric contraction. In addition, the free surface of the composite recycled aggregate exposed to ambient air exhibits a lower moisture content than the surrounding concrete, which promotes the migration and evaporation of water from the cement matrix. Although the increase in drying shrinkage agrees with previous studies on recycled plastic aggregates,^51^ the magnitude of the increase is lower than that reported for fully polymeric aggregates, again suggesting that the mineral fraction of CRA provides a partial restraint against shrinkage deformation.

Regarding compressive strength, the obtained results, which show a marked reduction with increasing levels of natural aggregate replacement, are consistent with the available literature. Previous studies have indeed reported a clear tendency toward reduced compressive strength when plastic aggregates, either fine or coarse, are incorporated into concrete, regardless of the nature of the recycled material.^6^

It is worth noting that the strength reductions measured in the present study, particularly when coarse CRA were used, are generally smaller than those reported for concretes incorporating fully plastic aggregates at comparable replacement ratios.^6,7^ This finding suggests that the rigid quartz particles embedded within the composite recycled aggregates partially compensate for the weak polymer–cement interface, thereby mitigating the loss of mechanical performance and supporting the potential use of coarse CRA in structural concrete applications. This interpretation is also supported by microstructural observations. Similar interfacial gaps have been reported by several authors investigating recycled plastic aggregates through SEM observations ,^6^ indicating that poor wettability is one of the main mechanisms responsible for the deterioration of the interfacial transition zone. However, unlike fully polymeric particles, the composite recycled aggregates investigated in the present study still exhibit localized mechanical interlocking provided by the quartz-rich phase, which likely contributes to the less severe mechanical degradation observed experimentally.

A tendency toward reduced stiffness in CRA concrete is consistent with the findings available in the literature referring to various types of plastic aggregates,^6,16^ which report a general decrease in elastic modulus compared to conventional concrete. For example, as reported by Gu et al.^6^ at a 20% replacement level of natural aggregates with plastic aggregates, a decrease in elastic modulus ranging from about 14% to 28% can be observed, largely consistent with the experimental results of the present study. This behavior can be mainly attributed to the lower elastic modulus of plastic aggregates; however, the weak bond between the cement matrix and the recycled aggregates contributes to this reduction as well. Nevertheless, when coarse CRA were used, the reduction in elastic modulus remained close to the lower bound of the ranges reported for fully plastic aggregates, further suggesting that the rigid quartz-rich mineral fraction partially compensates for the detrimental effect of the polymeric phase.

As regards tensile behavior, in the case of plastic aggregates, the scientific literature consistently confirms a general reduction in tensile strength,^6^ with a decreasing trend for increasing replacement ratio due to the above-described worsening of ITZ. Hence, the general reduction of tensile performance of CRA concrete can once again be explained by the presence of plastics components in the composite aggregates. However, as reported in some cases for plastics, their adverse impact on flexural and tensile strength appears to be less pronounced than that on compressive strength, especially for moderate replacement ratios of natural aggregates (around 20%).^6^ This is due to the ability of plastics to slow down the propagation of microcracks, resulting in concrete with increased ductility. This behavior, which was already reported in only some studies in the literature for plastic aggregates,^46,52^ is also confirmed by the fracture energy results of the present study, where CRA concrete exhibits even improved toughness compared to reference NA concrete, thanks to improved

All crack deflection and the higher deformability of the inclusions that enhance energy absorption during fracture. However, other studies have also reported a reduction in fracture energy, indicating that the influence of polymeric inclusions depends on their type, geometry, and interaction with the cement matrix.^22,53^

The present findings extend these observations to hybrid quartz–polymer recycled aggregates, suggesting that the combined presence of rigid mineral particles and deformable polymeric phases may contribute to achieving a more favorable balance between compressive strength, tensile/flexural strength, and fracture toughness than would be expected from polymeric inclusions alone.

Overall, although the mechanisms governing the behavior of CRA concrete share several similarities with those reported for concretes incorporating plastic aggregates, the present findings demonstrate that hybrid quartz–polymer composite recycled aggregates cannot be considered equivalent to conventional recycled plastic aggregates.

These findings also suggest that design approaches developed for conventional plastic aggregate concretes may not be directly applicable to hybrid quartz–polymer recycled aggregates, and that future mix design methodologies should explicitly account for the complementary mechanical roles of the polymeric and mineral phases rather than treating CRA as a homogeneous plastic aggregate.

Among the properties compared above, CRA concrete essentially occupies an intermediate position between conventional NA concrete and concrete containing recycled plastic aggregates. Compared with the latter, concrete with coarse CRA exhibits a less pronounced reduction in compressive strength and elastic modulus, bringing these properties closer to those of conventional concrete. In contrast, its tensile and fracture properties are comparable to, or in some cases better than, those reported for conventional concrete. This intermediate and nonuniform positioning is a characteristic of the quartz-polymer hybrid composition: the mineral fraction limits the losses in strength and stiffness typically caused by the polymer phase, while the polymer phase still contributes to the crack deflection and toughness effects associated with the plastic inclusions. In practice, this means that the mechanical performance and safety factors studied for either entirely mineral or entirely plastic recycled aggregates cannot be applied to CRA concrete, and the specific characterization of the mixture remains necessary until composition-based design rules are established.

### 6.3 Environmental Performance and Mechanical Trade-Off

From an environmental impact perspective, the comparison among the different mixes showed that the F15G30 mix achieves the greatest impact reduction across all EN 15804 ^43^ impact categories considered, with the exception of Water Use. Since cement dominates the overall impact (Figure 23), the benefits of substitution of NA with CRA are only marginally appreciable. Accordingly, the percentage difference with respect to the Climate Change impact of R0 amounts to 2 percentage points for F15 and G15, 4 percentage points for G30, and 6 percentage points for F15G30.

**Figure 23:**
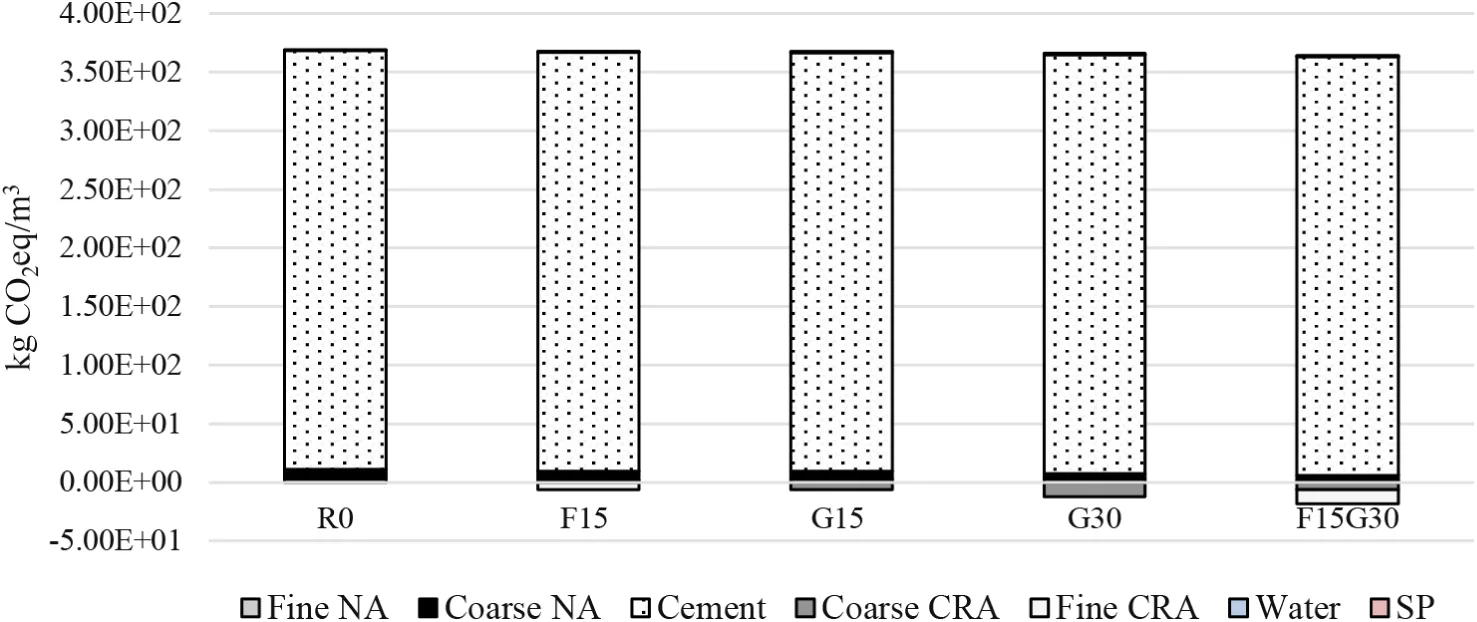
Contribution analysis related to Climate Change.

However, relevant impact reductions are achieved for categories such as Marine Eutrophication, where the effect of the avoided impact associated with the landfill disposal of the composite material incorporated in coarse and fine CRA can counterbalance the impact of cement (Figure 24). Overall, it can be stated that the circularity strategy applied to the mixes is effective in reducing the environmental burdens of concrete, but it leads to appreciable benefits only for certain impact categories.

**Figure 24:**
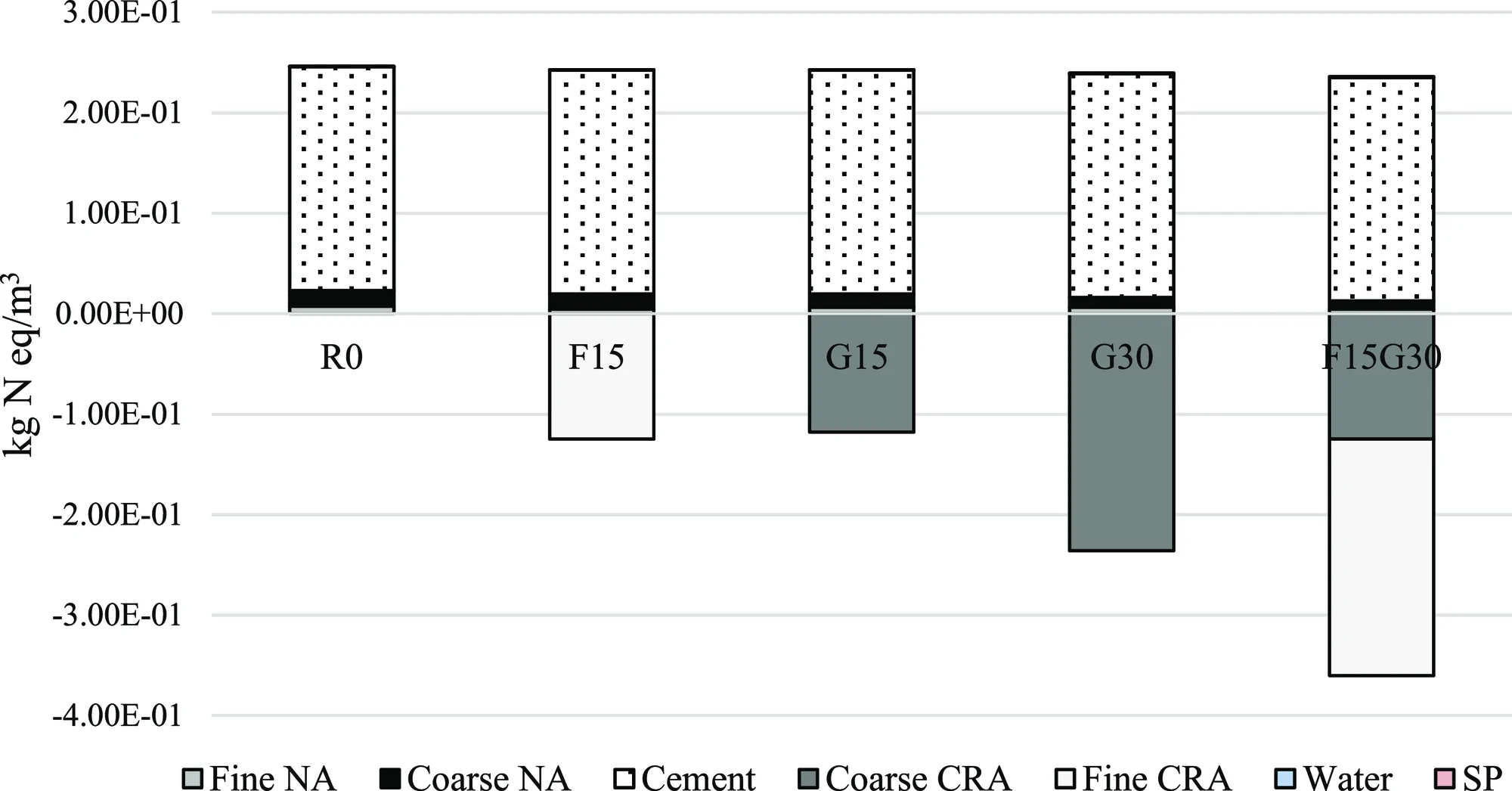
Contribution analysis related to Marine Eutrophication.

The results of the sensitivity analysis described in Section 5.5 provide additional insights into the robustness of the environmental assessment outcomes. By varying the modeling approach adopted for the substituted natural aggregates and using data sets representative of different geographical contexts, only marginal effects on the overall results associated with 1 m^3^ of concrete mix were observed. Furthermore, the environmental ranking of the different concrete mixes remained unchanged. In contrast, the modeling choices adopted for recycled aggregates were found to have a significant influence on the environmental results, particularly for impact categories that are more strongly affected by the avoided landfill credit, namely Marine Eutrophication, Land Use, and Water Use. Other impact categories, such as Climate Change, proved to be more robust to these modeling assumptions. Therefore, despite the limitations of the study related to the dependence of environmental results on methodological choices and to the assumption that the different concrete mixes are comparable in terms of their intended application, the findings suggest that the use of composite waste materials as a replacement for natural aggregates can represent a viable sustainability strategy under specific conditions.

It should be emphasized that the improvement in environmental performance, computed by using a volumetric functional unit (1 m^3^ of concrete) is accompanied by a progressive reduction in the mechanical properties as the replacement level of natural aggregates increases. Therefore, the selection of the replacement percentage should always be evaluated together with the corresponding mechanical performance, ensuring that the reduction in mechanical properties remains within acceptable limits for the intended application. Since the use phase was excluded from the present LCA and the comparison was performed on a volumetric functional unit, the scope of this study does not extend to the assessment of specific applications or service-life performance. Consequently, the environmental results should be interpreted within the boundaries of the adopted assessment framework and in conjunction with the mechanical requirements of the intended use. It is worth noting that integrated eco-mechanical approaches, in which environmental indicators are combined with the corresponding mechanical performance through eco-mechanical indexes, have been proposed in the literature^54^ to support the identification of optimal mix designs containing waste from both environmental and structural perspectives. Such approaches could represent a valuable extension of the present work, allowing the environmental benefits associated with recycled aggregate replacement to be directly balanced against the corresponding reduction in mechanical performance.

When analyzing the mechanical and environmental sustainability results together, three replacement regimes emerge. Below a coarse-grained replacement rate of approximately 15% (G15), the environmental benefit is modest but essentially free of mechanical trade-offs, making this regime the most justifiable for structural use.

When natural aggregate is replaced with coarse-grained CRA (G30) at a rate of between 15% and 30%, the environmental benefit roughly doubles, while mechanical losses remain within generally acceptable limits: therefore, the overall balance continues to favor substitution. Beyond this point, and whenever fine-grained CRA (F15, F15G30) is introduced, the additional environmental benefits are increasingly offset by mechanical performance losses, making these mixtures more suitable for nonstructural applications where environmental benefits can be achieved without the risk of compromising structural performance.

It is this interpretation based on performance regimes, rather than the discussion of individual impact categories outlined above, that ultimately determines what level of CRA content is justified for a given application.

## 7 Conclusions

This work investigates the effects of incorporating into concrete different particle size recycled aggregates—derived from composite material waste from sink manufacturing—on physical, mechanical and durability performance of concrete. The combined assessment performed provides a comprehensive picture of the potential and limitations of this waste valorization strategy, leading to the following main conclusions:

1) CRA replacement modifies several concrete properties, with effects depending on both replacement level and particle size.
2) Workability is not significantly affected by the presence of composite recycled aggregates, with measured values that are comparable to those of natural aggregate concrete. The maximum variation was observed in the mixture with 30% replacement of coarse aggregates, which shows an increase in the slump value of approximately 7%.
3) The use of composite recycled aggregates leads to a decrease in mixture density, which is more pronounced when fine recycled aggregates are used. In the case of hardened concrete, this reduction reaches up to 22% when a 30% of coarse and 15% of fine ones are replaced.
4) In terms of both total water absorption and water absorption under pressure, increasing the substitution rate of composite recycled aggregates leads to higher measured values. Nevertheless, the results remain compatible with structural concrete requirements, including sorptivity values.
5) Drying shrinkage values increase with higher percentages of aggregate replacement.
6) Compressive strength decreases when composite recycled aggregates are used in the concrete mix. In the mixture with the highest substitution level (30% replacement of coarse aggregates and 15% of fine aggregates), the strength reduction at 120 days is approximately 57%. It can be observed that the particle size of the composite recycled aggregates significantly influences the compressive strength: the average value measured at 120 days for the G15 specimens containing 15% coarse recycled aggregates is approximately 27% higher than that obtained for the F15 specimens containing the same percentage of fine recycled aggregates.
7) Tensile and flexural strength are less influenced than compressive strength by the replacement of natural aggregates with recycled aggregates. Moreover, the presence of coarse composite recycled aggregates positively influences the postcracking tensile behavior, resulting in enhanced fracture energy after cracking onset, with 9% and 4% higher values for specimens with 15% and 30% replacement of natural aggregates with respect to reference concrete.
8) The material stiffness in terms of elastic modulus decreases as the replacement percentage increases.
9) The environmental performance of the concrete mix improves as the replacement percentage increases, although the benefits are significant only for certain impact categories. Moreover, sensitivity analysis revealed that environmental results are sensitive to the modeling approach chosen for recycled aggregates. The environmental credit for avoided landfilling primarily affects impact categories related to Marine Eutrophication, Land Use, and Water Use, whereas results concerning Climate Change prove to be more stable.

The effects of composite material waste aggregate addition on physical, mechanical and durability properties of concrete can be attributed to several factors: a) the hydrophobic nature of the plastic compounds contained in composite aggregates, which limits cement hydration reactions near the surface of the aggregate, reducing the efficiency of the chemical bond between aggregate and cement paste, with consequent increased porosity, reduced mechanical strength, but enhanced toughness; b) the lower density of composite recycled aggregates compared to natural ones, which reduces concrete density; c) the lower elastic modulus of composite recycled aggregates, which influences not only the stiffness of concrete, but also the mechanism of crack propagation and energy absorption.

The results demonstrate that the production of eco-concrete with recycled aggregates derived from composite material waste containing plastic compounds is feasible. However, its recommended use should be limited to nonstructural elements—particularly when fine composite aggregates are employed—or to specific applications where improved flexibility or toughness is desirable. It should also be emphasized that the environmental comparison was performed using a volumetric functional unit (1 m^3^ of concrete), while the use phase was excluded from the system boundaries. Therefore, the environmental benefits should always be considered together with the corresponding mechanical performance, ensuring that the reduction in mechanical properties remains acceptable for the intended application.

Although the present results demonstrate the technical feasibility of the proposed approach, some aspects deserve further investigation before wider implementation. In particular, the long-term performance of concrete incorporating composite recycled aggregates under aggressive exposure conditions (e.g., freeze–thaw cycles, chloride ingress, carbonation, and sulfate attack) requires further investigation, particularly for potential structural applications. Furthermore, while SEM observations provided valuable insight into the characteristics of the interfacial transition zone (ITZ), further quantitative microstructural characterization (e.g., nanoindentation, microhardness mapping, or EDS line scanning) would improve the understanding of the bond mechanisms between the cement matrix and the composite recycled aggregates and their evolution over time. Finally, future studies should also consider application-specific performance requirements and alternative LCA frameworks based on functional equivalence and service life.

Overall, this study demonstrates a promising valorization pathway for composite material waste, contributing to landfill diversion, reduced consumption of virgin natural aggregates, more sustainable concrete production, and the transition toward a circular economy. Nevertheless, the proposed approach should be considered an application-specific solution, whose suitability depends on the required mechanical performance and service conditions, rather than a universally applicable replacement strategy for all concrete applications.

Beyond the specific results obtained for the investigated mixtures, the present study highlights the importance of adopting an integrated evaluation framework for the development of circular concrete incorporating waste-derived materials. By combining chemical characterization, mechanical testing, microstructural observations, and life cycle assessment, a more comprehensive understanding of both the technical feasibility and the environmental implications of these innovative materials can be achieved. Such a multidisciplinary approach is essential for supporting the responsible implementation of waste-derived constituents in concrete and represents one of the main contributions of the present work, particularly considering that studies integrating these complementary perspectives within a single framework remain relatively limited.

## Supplementary Material



## Notes

Additional experimental results and Life Cycle Assessment (LCA) results—supplementary figures and tables (PDF)
