# Supplementary material for: Mechanical and Environmental Performance of Circular Concrete with Recycled Aggregates from Composite Material Waste
Source: ACS Omega. 2026 Oct 1;11(40):60920–40. doi: 10.1021/acsomega.6c04730 (PMC13670260; doi:10.1021/acsomega.6c04730)
Supplement: Supplementary file 1 [file ao6c04730_si_001.pdf]

MECHANICAL AND ENVIRONMENTAL PERFORMANCE OF CIRCULAR CONCRETE WITH RECYCLED AGGREGATES FROM COMPOSITE MATERIAL WASTE

Patrizia Bernardi[a], Federico Pagliari[a], Alice Sirico[a], Arianna Paini[b], Corrado Sciancalepore[b], Daniel Milanese[b], Giuseppe Vignali[b], Beatrice Belletti[a]

[a] Department of Engineering and Architecture, University of Parma, Parco Area delle Scienze, 181/A, 43124 Parma, Italy

[b] Department of Engineering for Industrial Systems and Technologies, University of Parma, Parco Area delle Scienze, 181/A, 43124 Parma, Italy

*Corresponding author: Alice Sirico

E-mail address: alice.sirico @unipr.it

SUPPORTING INFORMATION: additional experimental results and life cycle assessment (LCA) results – supplementary figures and tables (PDF).

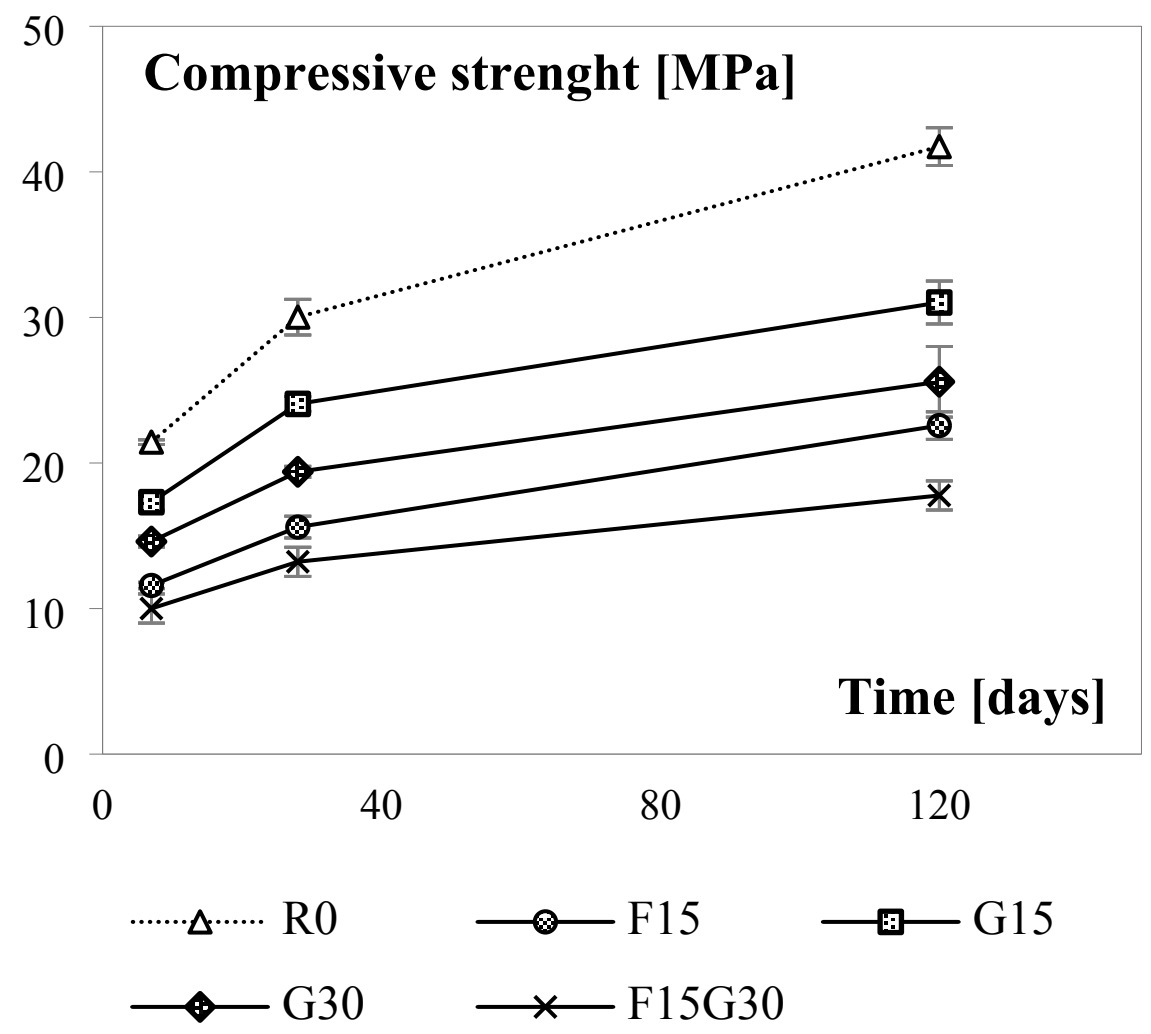

Figure S1. Development of compressive strength over time (with the corresponding standard deviation) for concrete mixes containing composite recycled aggregates (F15, G15, G30 and F15G30) and reference concrete (R0).

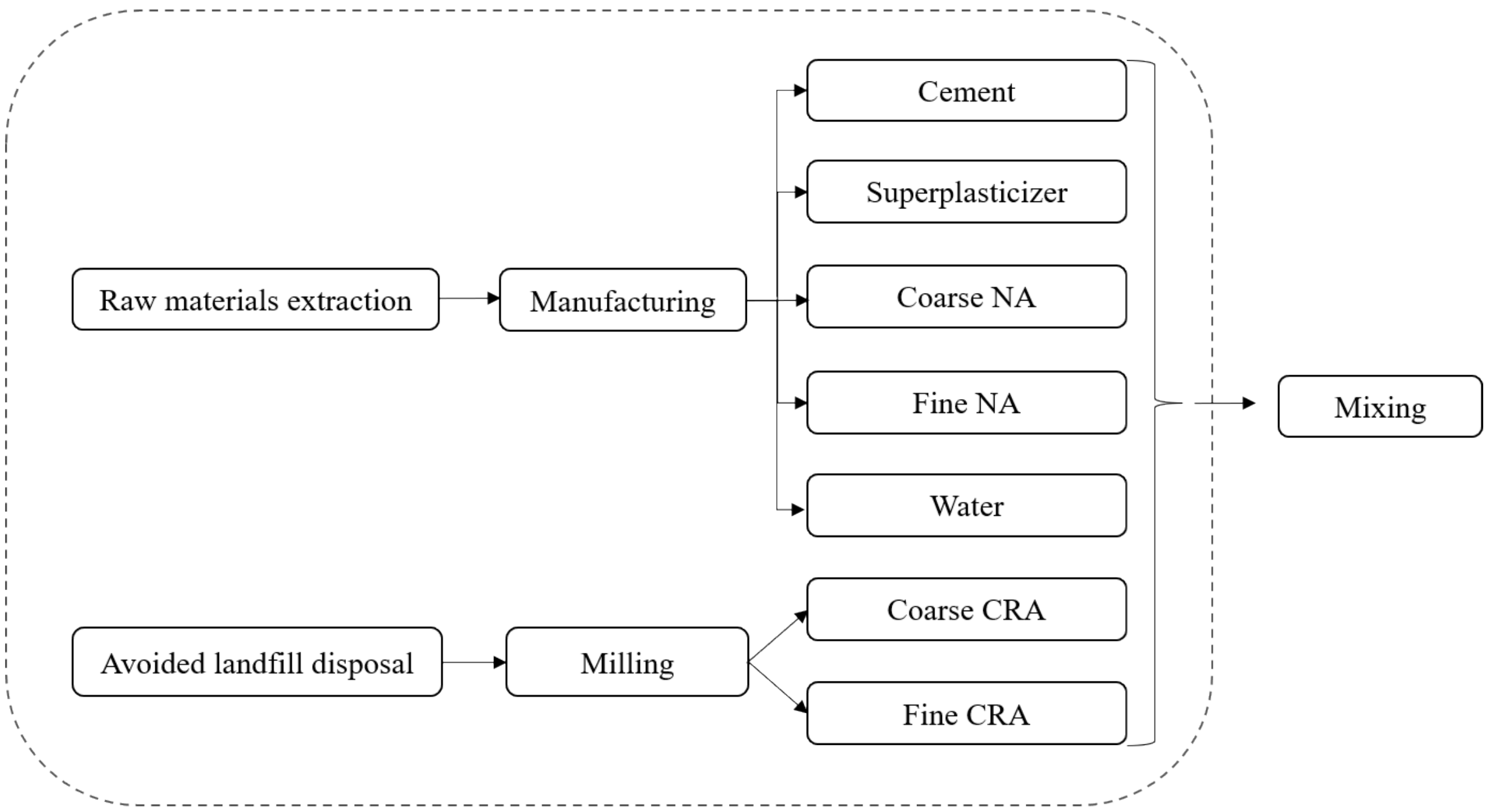

Figure S2. System boundaries of LCA analysis.

Impact category	Unit	Coarse CRA	Fine CRA
Acidification	mol H+ eq	-8.61E-05	-7.79E-05
Climate change	kg CO_2 eq	-3.32E-02	-3.21E-02
Climate change - Biogenic	kg CO_2 eq	-7.38E-05	-7.31E-05
Climate change - Fossil	kg CO_2 eq	-3.31E-02	-3.20E-02
Climate change - Land use and LU change	kg CO_2 eq	-1.18E-07	1.39E-06
Eutrophication, marine	kg N eq	-6.45E-04	-6.44E-04
Eutrophication, freshwater	kg P eq	-1.10E-05	-1.04E-05
Eutrophication, terrestrial	mol N eq	-2.80E-04	-2.67E-04
Land use	Pt	-5.64E-01	-5.60E-01
Ozone depletion	kg CFC11 eq	-2.16E-10	-1.43E-10
Photochemical ozone formation	kg NMVOC eq	-1.10E-04	-1.17E-04
Resource use, fossils	MJ	-2.14E-01	-2.01E-01
Resource use, minerals and metals	kg Sb eq	8.66E-08	1.60E-07
Water use	m^3 depriv.	9.45E-02	9.55E-02

Table S1. Environmental impacts related to 1 kg of coarse and fine CRA.

Impact category	Unit	Milling	Landfill disposal	Coarse CRA (Milling-Landfill disposal)
Acidification	mol H+ eq	1.19E-05	9.80E-05	-8.61E-05
Climate change	kg CO_2 eq	1.54E-03	3.47E-02	-3.32E-02
Climate change - Biogenic	kg CO_2 eq	1.08E-06	7.49E-05	-7.38E-05
Climate change - Fossil	kg CO_2 eq	1.54E-03	3.47E-02	-3.31E-02
Climate change - Land use and LU change	kg CO_2 eq	2.18E-06	2.30E-06	-1.18E-07
Eutrophication, marine	kg N eq	1.77E-06	6.47E-04	-6.45E-04
Eutrophication, freshwater	kg P eq	9.50E-07	1.20E-05	-1.10E-05
Eutrophication, terrestrial	mol N eq	1.87E-05	2.99E-04	-2.80E-04
Land use	Pt	6.71E-03	5.71E-01	-5.64E-01
Ozone depletion	kg CFC11 eq	1.05E-10	3.20E-10	-2.16E-10
Photochemical ozone formation	kg NMVOC eq	6.82E-06	1.17E-04	-1.10E-04
Resource use, fossils	MJ	1.92E-02	2.34E-01	-2.14E-01
Resource use, minerals and metals	kg Sb eq	1.07E-07	2.01E-08	8.66E-08
Water use	m^3 depriv.	1.49E-03	-9.30E-02	9.45E-02

Table S2. Contribution of the milling process and avoided impacts of landfill to the overall impact of 1 kg of coarse CRA.

**Impact category**	**Unit**	**R0**	**F15**	**G15**	**G30**	**F15G30**
Acidification	mol H+ eq	8.20E-01	7.97E-01	7.94E-01	7.68E-01	7.43E-01
Climate change	kg CO_2 eq	3.69E+02	3.62E+02	3.62E+02	3.54E+02	3.46E+02
Climate change - Biogenic	kg CO_2 eq	6.05E+00	6.02E+00	6.01E+00	5.97E+00	5.93E+00
Climate change - Fossil	kg CO_2 eq	3.63E+02	3.56E+02	3.55E+02	3.48E+02	3.40E+02
Climate change - Land use and LU change	kg CO_2 eq	6.80E-02	6.78E-02	6.73E-02	6.65E-02	6.61E-02
Eutrophication, marine	kg N eq	2.46E-01	1.19E-01	1.25E-01	3.55E-03	-1.25E-01
Eutrophication, freshwater	kg P eq	3.82E-02	3.60E-02	3.59E-02	3.37E-02	3.15E-02
Eutrophication, terrestrial	mol N eq	2.80E+00	2.71E+00	2.71E+00	2.61E+00	2.52E+00
Land use	Pt	5.68E+02	4.33E+02	4.32E+02	2.94E+02	1.55E+02
Ozone depletion	kg CFC11 eq	1.58E-06	1.53E-06	1.51E-06	1.45E-06	1.39E-06
Photochemical ozone formation	kg NMVOC eq	8.07E-01	7.75E-01	7.75E-01	7.42E-01	7.09E-01
Resource use, fossils	MJ	1.63E+03	1.57E+03	1.57E+03	1.51E+03	1.44E+03
Resource use, minerals and metals	kg Sb eq	4.36E-04	4.59E-04	4.39E-04	4.41E-04	4.61E-04
Water use	m3 depriv.	3.52E+01	5.31E+01	5.21E+01	6.91E+01	8.71E+01

Table S3. Comparative impact results referred to 1 m3 of each concrete mix
